# Dynamic states of eIF6 and SDS variants modulate interactions with uL14 of the 60S ribosomal subunit

**DOI:** 10.1093/nar/gkac1266

**Published:** 2023-01-18

**Authors:** Jonah Elliff, Aparna Biswas, Poonam Roshan, Sahiti Kuppa, Angela Patterson, Jenna Mattice, Mathivanan Chinnaraj, Ryan Burd, Sarah E Walker, Nicola Pozzi, Edwin Antony, Brian Bothner, Sofia Origanti

**Affiliations:** Department of Biological Sciences, Marquette University, Milwaukee, WI 53233, USA; Department of Immunology, The University of Iowa, Iowa City, IA 52242, USA; Department of Biology, Saint Louis University, St. Louis, MO 63103, USA; Department of Biology, Saint Louis University, St. Louis, MO 63103, USA; Department of Biochemistry and Molecular Biology, Saint Louis University School of Medicine, MO 63104, USA; Department of Chemistry and Biochemistry, Montana State University, Bozeman, MT 59717, USA; Department of Chemistry and Biochemistry, Montana State University, Bozeman, MT 59717, USA; Department of Biochemistry and Molecular Biology, Saint Louis University School of Medicine, MO 63104, USA; Department of Biological Sciences, Marquette University, Milwaukee, WI 53233, USA; Department of Biological Sciences, State University of New York, Buffalo, NY 14260, USA; Department of Biochemistry and Molecular Biology, Saint Louis University School of Medicine, MO 63104, USA; Department of Biochemistry and Molecular Biology, Saint Louis University School of Medicine, MO 63104, USA; Department of Chemistry and Biochemistry, Montana State University, Bozeman, MT 59717, USA; Department of Biology, Saint Louis University, St. Louis, MO 63103, USA

## Abstract

Assembly of ribosomal subunits into active ribosomal complexes is integral to protein synthesis. Release of eIF6 from the 60S ribosomal subunit primes 60S to associate with the 40S subunit and engage in translation. The dynamics of eIF6 interaction with the uL14 (RPL23) interface of 60S and its perturbation by somatic mutations acquired in Shwachman–Diamond Syndrome (SDS) is yet to be clearly understood. Here, by using a modified strategy to obtain high yields of recombinant human eIF6 we have uncovered the critical interface entailing eight key residues in the C-tail of uL14 that is essential for physical interactions between 60S and eIF6. Disruption of the complementary binding interface by conformational changes in eIF6 disease variants provide a mechanism for weakened interactions of variants with the 60S. Hydrogen–deuterium exchange mass spectrometry (HDX-MS) analyses uncovered dynamic configurational rearrangements in eIF6 induced by binding to uL14 and exposed an allosteric interface regulated by the C-tail of eIF6. Disrupting key residues in the eIF6–60S binding interface markedly limits proliferation of cancer cells, which highlights the significance of therapeutically targeting this interface. Establishing these key interfaces thus provide a therapeutic framework for targeting eIF6 in cancers and SDS.

## INTRODUCTION

Several *trans*-acting factors coordinate the assembly and maturation of the ribosomal subunits (1–4). Eukaryotic translation initiation factor-6 (eIF6) is one such essential factor that is crucial for the biogenesis and maturation of 60S subunits (5–8). It also functions as a 60S silencing or anti-association factor that sterically inhibits 60S association with the 40S subunit by disrupting inter-subunit bridges (9–14). Release of eIF6 from 60S is thus critical to facilitate interactions with 40S and to permit the formation of translationally competent 80S monosomes (9–14).

Impaired function of eIF6 contributes to the underlying pathology of certain cancers and inherited ribosomopathies: Shwachman–Diamond Syndrome (SDS) and a subset of pediatric T-cell acute lymphoblastic leukemia (15–18). eIF6 is overexpressed in several cancers including colon, ovarian and breast cancers and enhanced expression is correlated with a poor prognosis (19–26). However, it remains unclear as to how overexpression of eIF6 drives tumorigenesis. Partial loss of eIF6 limits the transformation efficiency of oncogenic H-Ras12V mutant and oncogenic Myc. A reduction in eIF6 levels in malignant pleural mesotheliomas and in Myc-induced lymphomas markedly impairs cell growth by inhibiting protein synthesis rates (20–22). Since haploinsufficiency of eIF6 delays tumorigenesis without markedly affecting normal growth, it presents eIF6 as a viable therapeutic target with potentially minimal side effects (21).

Disrupting eIF6 activity has thus been proposed to be a therapeutic strategy for the treatment of certain cancers and SDS. This therapeutic strategy is especially significant for SDS that is predominantly driven by biallelic mutations in the Shwachman–Bodian–Diamond syndrome (*SBDS*) factor (16,18,27). SDS is an inherited disorder associated with bone marrow failure, exocrine pancreatic insufficiency, skeletal deformities, developmental defects, and a predisposition to myelodysplastic syndrome (MDS) and acute myeloid leukemia (28,29). Germline mutations in *SBDS* and *elongation factor like GTPase-1* (*EFL1*) hinder 60S maturation and prevent the release of eIF6 from nascent 60S leading to impaired subunit joining and reduced translational fitness (16–18,28,30). It is likely that the release of eIF6 from recycled 60S subunits post-termination is also disrupted by SBDS deficiency (31). Recent studies have detected somatic mutations in *EIF6* in the hematopoietic cells of SDS patients that either reduced eIF6 expression or weakened interactions of eIF6 with 60S (32,33). Acquisition of such *EIF6* mutations present a compensatory mechanism that rescue the ribosomal and translational defect of SBDS deficient cells (32,33). Thus, these studies highlight the importance of targeting eIF6 activity for a better prognosis for SDS patients either by genetic compensation or by therapeutic means.

**Figure 1.**
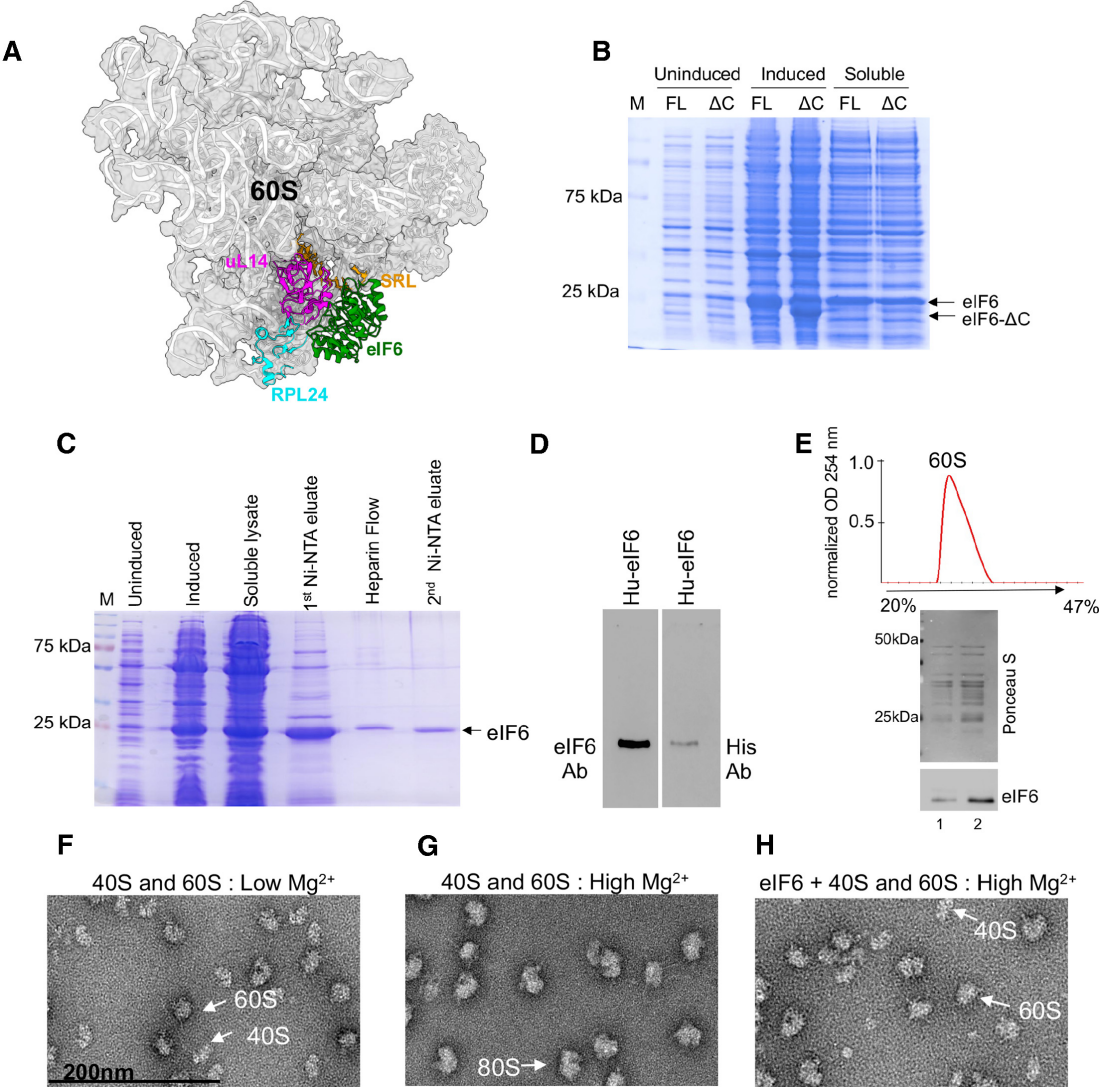
Purification of recombinant human eIF6. (**A**) Cryo-EM structure of human eIF6 bound to 60S (PBD code: 5AN9). eIF6 (green), uL14 (RPL23) (magenta), RPL24 (blue) and sarcin-ricin loop (SRL) (orange) are highlighted. (**B**) Representative Coomassie-stained gel shows induction and solubility of full-length (FL) and C-terminal deletion mutant of eIF6 (eIF6-ΔC). (**C**) Representative Coomassie-stained gel shows eIF6 protein at various stages of protein purification process using affinity chromatography. (**D**) Western blot analysis of purified eIF6. Recombinantly purified human eIF6 has intact N- and C-termini as protein is detected by anti-His antibody (Santa Cruz Biotechnology) targeted to the N-terminus and anti-eIF6 antibody (Cell Signaling) targeted to the C-terminus. (**E**) Ribosome profile (top) shows 60S peak and the western blot (below) shows proteins extracted from the 60S fractions. Data is representative of three independent replicates. Lanes 1 and 2 depict two different concentrations of 60S fraction. Blots were stained with Ponceau S to detect 60S ribosomal proteins and probed with anti-eIF6 antibody to determine co-elution of eIF6 with the 60S fraction. (**F**) Negative EM (magnified) images depict 60S and 40S subunits incubated in low Mg^2+^ buffer. (**G**) Negative EM (magnified) images depict the association of 60S and 40S subunits incubated in high Mg^2+^ buffer. (**H**) Negative EM (magnified) images depict 60S and 40S subunits incubated with eIF6 in high Mg^2+^ buffer.

A potent therapeutic strategy to disrupt eIF6 function is to block the critical interactions between eIF6 and 60S. However, to pursue such a targeted approach, there is a need to identify and experimentally interrogate the contributions of the residues in the binding pocket that are critical for eIF6 and 60S interactions. Structural studies show that eIF6 directly associates with uL14 (RPL23) and is proximal to the Sarcin-Ricin Loop (SRL) of the 28S rRNA and RPL24 (eL24) of 60S (Figure 1A) (11,34,35). One of the challenges towards performing extensive biophysical and biochemical characterization of the 60S-binding interactions is the inability to obtain sufficient quantities of full-length human eIF6 (36). Here, we describe a strategy to obtain milligram quantities of active full-length eIF6 by co-expression of the trigger factor (TF) chaperone. This development enabled us to probe eIF6 interactions with uL14 through detailed biophysical characterization. Using surface plasmon resonance (SPR), hydrogen-deuterium exchange mass spectrometry (HDX-MS), and cellular analyses we have identified residues in eIF6 and in the C-terminus of uL14 that are critical for eIF6–60S interactions. Our results show for the first time a dynamic transition state of eIF6 upon binding to uL14 that entails conformational changes in regions that are disrupted by SDS mutations. A summary of all the key eIF6 residues mentioned in this study and the associated mutations are listed in Supplementary Table S1.

The C-tail of eIF6 is highly conserved in higher eukaryotes. Several phosphoproteomic studies including our study captured multisite phosphorylation of the C-tail especially at S235, S239 and S243 sites (9,20,37–42). We found that eIF6 is critical for cells to adapt to starvation, which is regulated by phosphorylation of the C-tail (37). This role of eIF6 is akin to the ribosomal silencing factor- RsfS (RsfA) in bacterial cells that binds to the same uL14 (rplN) interface in the large 50S subunit and is critical for cells to limit global translation to adapt to nutrient-deprivation (43). Intriguingly, our HDX-MS analyses captured dynamic changes in the C-terminus of eIF6 upon binding to uL14 despite the absence of direct contacts between the C-terminus of eIF6 and uL14 in structural studies. Previously, phosphorylation of the S235 site was proposed to release eIF6 from the 60S (9). However, the mechanism for this allosteric mode of regulation has remained elusive. Our circular dichroism (CD) studies show that the addition of negative charges at conserved sites of phosphorylation in the C-tail markedly influence eIF6 conformation and could explain the molecular basis for allosteric regulation of eIF6–60S interactions by the C-tail. In addition, CD analyses have uncovered the influence of the predominant N106S mutation and the key Y151 residue on the overall secondary structure of eIF6. Based on these analyses, we selectively targeted the Y151 and N106 residues and show for the first time that perturbing a residue in the eIF6–60S interface markedly affects cancer cell proliferation through disruption of binding to uL14. Thus, the key residues of interaction identified in both uL14 and eIF6 in this study will aid in the design of small molecule inhibitors that can be docked in this critical interaction interface. This will enable the development of novel therapeutics that target eIF6 for the treatment of cancers and SDS.

## MATERIALS AND METHODS

### Cloning and site-directed mutagenesis of eIF6

Codon-optimized full-length human *EIF6* (IDT Inc.) was cloned into MCS1 of pRSF-DUET-1 vector. Site specific mutations were introduced using Q5-site directed mutagenesis (New England Biolabs) and primers were designed using NEBaseChanger program and were synthesized by Integrated DNA Technologies. Codon-optimized full-length *TIF6* (Genscript Inc.) was cloned into MCS1 of pRSF-DUET-1 plasmid. List of primers used are indicated in Supplementary Table S2.

### Purification of recombinant full-length human eIF6

Recombinant full-length human eIF6 was co-expressed with trigger factor (TF) in *E. coli* BL21 (DE3) cells. pRSF-DUET-1-*EIF6* construct was used to express eIF6 and the pTf16 (Takara Biosciences) construct was used to express the TF chaperone. 1–4 l cultures were grown at 37°C in LB media supplemented with 50 μg/ml kanamycin, 34 μg/ml chloramphenicol, and 2 mg/ml l-arabinose (to induce chaperone expression). When cultures reached OD_600_ of 0.4 to 0.5, eIF6 expression was induced with 0.4 mM IPTG and cultures were shaken for 20 h at 20°C. Cells were harvested by centrifugation at 4000 rpm for 20 min. For cells collected from 1 l cultures, cells were resuspended in 40 ml of lysis buffer (50 mM Tris–HCl, pH 8.0, 300 mM NaCl, 10 mM imidazole, 1 mM β-mercaptoethanol, and 1 mM PMSF). In addition, 5× protease inhibitor cocktail (PIC) (Sigma-P2714), 2 μg/ml DNase, 20 μg/ml RNase and 1 mg/ml lysozyme were added to the resuspended cells. Cells were lysed by stirring for 30 min at 4°C, followed by flash freezing in liquid N_2_ and thawing in a water bath at room temperature (RT). The freeze-thaw cycle was repeated twice, followed by Dounce homogenization at 4°C. Lysate was clarified at 17 000 rpm (rotor ID: JA 25.50) for 1 h at 4°C. All purification steps indicated below were performed at 4°C.

The clarified lysate was applied onto a Ni^2+^-nitrolotriacetic acid (NTA) column (gravity flow) packed with 2.5 ml bed volume (BV) of agarose resin per 1 l culture. Non-specifically bound proteins were washed off with 20× bed volume of lysis buffer followed by two washes with 2× BV of wash buffer (50 mM Tris pH 8.0, 1 M NaCl, 60 mM imidazole, 1 mM β-mercaptoethanol, 1× PMSF and 3× PIC). Protein was eluted using 5× BV of elution buffer (50 mM Tris pH 8.0, 300 mM NaCl, 300 mM imidazole, 1 mM β-mercaptoethanol, 1× PMSF and 5× PIC). The eluate was collected as five equal fractions and assessed by SDS-PAGE analysis. Fractions containing eIF6 were pooled, diluted 6-fold using H^0^ buffer (50 mM Tris–Cl pH 8.0 and, 1mM β-mercaptoethanol), and loaded onto to a 5 ml HiTrap-Heparin prepacked column (Cytiva Inc.) at 3 ml/min. Before loading the protein, the Heparin column was washed with 6× column volume (CV) of H^50^ buffer (50 mM Tris–Cl pH 8.0, 1 mM β-mercaptoethanol and 50 mM NaCl). eIF6 does not bind to the Heparin column under these conditions and thus flows through. eIF6 in the flow-through fraction was loaded onto a new Ni^2+^-NTA gravity flow column and washed with lysis buffer and wash buffer as described above. eIF6 was eluted using 5X BV of elution buffer (50 mM Tris pH 8.0, 300 mM NaCl, 300 mM imidazole, 1 mM β-mercaptoethanol, 1× PMSF and 5× PIC). Five fractions of equal volume were collected and analyzed by SDS-PAGE. Fractions containing eIF6 were pooled and concentrated using an Amicon Ultra 10 kDa centrifugal concentrator and centrifuged at 4000 rpm for 10 min. To avoid mild precipitation, final volume of the concentrate from 1 l culture was maintained around 500 μl. Concentrated protein was dialyzed overnight against final storage buffer (50 mM HEPES pH 7.5, 200 mM NaCl, 5 mM β-mercaptoethanol, and 10% glycerol) and briefly spun down. Small aliquots were flash frozen in liquid N_2_ and stored at −80°C. (Note: full-length human eIF6 is prone to degradation if protein is frozen and thawed multiple times and thus, more than two freeze-thaw cycles should be avoided. After being thawed, full-length eIF6 does not tolerate re-concentration using centrifugal concentrators or overnight dialysis, at least in the buffer conditions that we tested. These processes result in either mild degradation or mild precipitation of eIF6.) Mutant eIF6 proteins were overproduced and purified using the same procedure. eIF6 concentration was measured using ϵ_280_ 11 460 M^−1^ cm^−1^. To identify the optimal co-chaperone combinations that enhance eIF6 solubility, pRSFDUET-1-*EIF6* plasmid was co-transformed with pG-KJE8 expressing five bacterial chaperones (5-Ch: DnaK-DnaJ-GrpE, GroES, GroEL) or pG-TF2 expressing 3 bacterial chaperones (3-Ch: GroES, GroEL, TF) (Takara Biosciences) in BL21 (DE3) cells. Recombinant human eIF6 were purified as described above. For these experiments, an additional purification step using size-exclusion chromatography on a Superdex S200 column (GE Healthcare) was included to remove all chaperones. For comparison of eIF6 expression in the soluble fraction in the absence and presence of the TF chaperone, cells were transformed with pRSFDUET-1-*EIF6* plasmid only or co-transformed with the pTf16 plasmid as described above. Equal volumes of uniduced, induced and soluble lysate was subjected to SDS-PAGE and assayed by Commassie staining or by western blotting using anti-eIF6 antibody (sc-390441, Santa Cruz Biotechnology).

### Surface plasmon resonance (SPR)

SPR analyses were carried out using a BIAcore S200 instrument (GE-Healthcare). eIF6 (50 μg/ml) was solubilized in 20 mM ammonium acetate pH 4.0 and immediately immobilized on a CM5 sensor chip (4913 Response Units [RU] using NHS/EDC chemistry). After baseline stabilization, titrations were performed by injecting increasing concentrations (0–500 μM, 1:2 dilutions) of uL14-WT (VAKECADLWPRIASNAGSIA), the site-specific mutant peptides, or the uL14-ΔC (VAKECADLWPRI) peptide, solubilized in running buffer (20 mM HEPES pH 7.4, 150 mM NaCl, 0.01% BSA, and 0.002% Tween 20). At the end of each titration, a solution of 20 mM HEPES pH 7.4 and 1.5 M NaCl was used to regenerate the chip. The flow rate was 25 μl/min. Peptides were solubilized in water and their concentration determined at 280 nm using the molar extinction coefficient ϵ_280_ = 5500 M^−1^ cm^−1^. To ensure reproducibility, each experiment was repeated three times. Sensograms were corrected for baseline.

### Chemical cross-linking of eIF6 to uL14 peptide

Cross-linking reactions were performed in 50 mM HEPES pH 7.5, and 200 mM NaCl. eIF6 (24 μM final concentration) was mixed with the uL14 peptide (0.7 mM final concentration) and crosslinker was added. 1,8-bismaleimido-diethyleneglycol (BM(PEG)2) was added to a final concentration of 3 mM and the reaction was quenched at 15 min and 1 h with the addition of dithiothreitol (DTT, final concentration 48 mM). Quenched crosslinking reactions were then run on a Bio-Rad precast 4–20% gel (Mini-Protean TGX) at 150 V for 45 min. The gel was stained overnight with GelCode Blue Safe Protein Stain (Thermo-Fisher) and destained with deionized water. The destained gel was then subjected to a standard in gel digestion (44) and the excised band digest was analyzed using liquid chromatography–mass spectrometry (LC–MS). LC–MS was performed on a maXis Impact UHR-QTOP instrument (Bruker Daltonics) as previously described (45). Peptides were identified using SearchGUI v3.3.16 coupled to PeptideShaker v1.16.42 (CompOmics) (46,47). Cross-linked tryptic peptides were identified using the mass of the tryptic uL14 peptide containing the crosslinkable residue as a modification and searching against the eIF6 protein sequence.

### HDX-MS of eIF6 and eIF6 bound to the uL14 peptide

eIF6 alone (174 μM) or eIF6 (87 μM) mixed with uL14 peptide (3.5 mM) were diluted 1:10 into deuterated buffer (50 mM HEPES, 200 mM NaCl, pD 7.5). At each time point (30 s, 3 min, 30 min, 3 h and 24 h), 10 μl of the HDX reaction were removed and quenched by diluting 1:6 into 0.75% formic acid (FA, Sigma), and digested for two min with porcine pepsin (0.25 mg/ml, Sigma) and vortexed every 30 seconds. Digested samples were then flash frozen and stored in liquid N_2_ until LC–MS analysis. LC–MS was carried out as described previously (48). Before MS analysis, LC was performed on a 1290 UPLC series chromatography stack (Agilent Technologies), and peptides were separated on a reverse phase column (Phenomenex Onyx Monolithic C18 column, 100 × 2 mm) at 1°C using a flow rate of 400 μl/min. The following conditions were used: 1.0 min, 5% B; 1.0−9.0 min, 5−45% B; 9.0−11.8 min, 45–95% B; 11.80−12.0 min, 5% B; solvent A = 0.1% FA (Sigma) in water (Thermo-Fisher) and solvent B = 0.1% FA in acetonitrile (ACN, Thermo-Fisher). MS data acquisition was carried out on a 6538 UHD Accurate-Mass QTOF LC/MS with the following settings: nebulizer set to 3.7 bar, drying gas at 8.0 l/min, drying temperature at 350°C, and capillary voltage at 3.5 kV. Data was acquired at 2 Hz s^−1^ over the scan range 50−1700 *m*/*z* in positive mode. Data analysis was carried out as previously described (49,50) using MassHunter Qualitative Analysis (Agilent Technologies), Peptide Analysis Worksheet (PAWs, ProteoMetrics LLC), SearchGUI v3.3.16, PeptideShaker v1.16.42, HDExaminer v 2.5.1 (Sierra Analytics), and visualized using UCSF Chimera (51). To assay for degradation or aggregation of eIF6, reactions were set-up for HDX-MS as described above except one-tenth of the reactants were used. Samples were incubated for 24 h at 4°C. Samples were briefly centrifuged to eliminate any settled aggregates or precipitates and equal volumes of the samples were collected from the top and denatured in 1× Laemmli sample buffer and subjected to SDS-PAGE analysis. No loss of sample was observed after centrifugation to eliminate aggregates indicating that there was negligible aggregation.

### Western blotting

For western blot analysis, proteins were resolved by SDS-PAGE and transferred onto nitrocellulose membranes (0.45 μm; Bio-Rad Laboratories). Membranes were blocked in 5% non-fat dry milk diluted in Tris-buffered saline with 0.1% Tween (TBS-T) buffer. The following primary antibodies, diluted in TBS-T buffer, were used: anti-eIF6 (1:1000, overnight, D16E9, Cell Signaling), anti–His (1:1000, overnight, sc-8036, Santa Cruz Biotechnology). Anti-mouse or anti-rabbit HRP-conjugated secondary antibodies (1:30 000, 1 h, Jackson Immunoresearch) were also used. For Ponceau S staining, membranes were rinsed in ultrapure water and stained with Ponceau S dye and de-stained with a brief rinse in ultrapure water. Gels and blots were imaged using iBright FL1500 imaging system (Fisher Scientific).

### Sucrose density gradient fractionation

Eukaryotic 60S (yeast) was purified as described previously (52). 40 pmol of eIF6 was mixed with 50 pmol of 60S in final volume of 10–20 μl resuspension buffer (20 mM HEPES pH 7.3, 100 mM KCl, 5% glycerol, 1 mM DTT, and 1 mM MgCl_2_). Reaction mixtures were incubated at 30°C for 5 min. Samples were loaded onto 20–47% sucrose gradient prepared as described before (37,53). Gradients were centrifuged at 35 000 rpm for 2 h 40 min using a SW41Ti rotor (Beckman). Gradients were fractionated using a Brandel (UV) gradient fractionation system and absorbance as described previously (37,53), and absorbance was monitored at 254 nm. Fractions corresponding to the 60S absorbance peak were collected and subjected to trichloroacetic acid (TCA)/acetone precipitation. Precipitated proteins were analyzed by western blotting as described above.

### Purification of human 60S and 40S ribosomal subunits

60S and 40S ribosomal subunits were purified from adherent HeLa cells as described previously with a few modifications (54,55). HeLa cells (ATCC) were maintained in DMEM medium (Gibco) supplemented with 10% fetal bovine serum (FBS), and 100 units/ml penicillin and 100 μg/ml streptomycin (Gibco). Cells were grown to 70–80% confluency in 15–20 of 150 mm dishes. Cells were washed twice in ice-cold phosphate buffered saline (PBS). Plates were briefly tilted to collect residual PBS, which was removed by aspiration. All further steps described below were carried out using RNase free tips and tubes, and buffers were made in nuclease-free ultra-pure water. Cells were scraped in lysis buffer (15 mM Tris–Cl pH 7.5, 300 mM NaCl, 7.5 mM MgCl_2_, 2 mM DTT, 1% Triton-X 100, 1 mg/ml heparin and 0.5× cOmplete Mini EDTA-free protease inhibitor cocktail [Roche]). Lysates were rocked at 4°C for 10 min followed by centrifugation at 10 000 rpm at 4°C for 10 min. The supernatant (∼4.5 ml) was layered onto 4 ml of ice-cold 30% sucrose cushion made just before use (20 mM Tris×Cl pH 7.5, 500 mM KCl, 30% w/v RNase free sucrose, 2 mM DTT, 10 mM MgCl_2_ and 0.1 mM EDTA pH 8.0) in centrifuge tubes (Beckman Coulter, catalog# 355630). Ribosomes were pelleted by centrifugation at 63 000g for 16–19 h at 4°C using a Type 80 Ti rotor (Beckman Coulter). For all ultracentrifugation steps indicated here, acceleration was set at Max and deceleration was set at Coast (no brake) setting (Beckman Coulter-Optima XPN 100 ultracentrifuge). Ribosome pellets were resuspended in 750 μl of resuspension buffer (20 mM Tris–Cl pH 7.5, 500 mM KCl, 7.5% w/v RNAse free sucrose, 2 mM MgCl_2_, 75 mM NH_4_Cl, 2 mM puromycin [Fisher Scientific], and 2 mM DTT) by gentle pipetting. The sticky pellet was gently scraped with a pipette tip, followed by gentle pipetting for 10min on ice to completely dissolve the pellet and till no chunks were visible. Ribosome pellets were then incubated at 4°C for 1 h to facilitate 60S and 40S subunit separation. Solution was mixed by gentle pipetting and then incubated at 37°C for 1 h. The solution was layered onto a linear 10–30% sucrose gradient (20 mM Tris–Cl pH 7.5, 500 mM KCl, w/v RNase-free sucrose and 6 mM MgCl_2_). Step gradients were prepared by freezing individual layers in liquid nitrogen in centrifuge tubes (Beckman Coulter-catalog#326823, maximum volume of 38 ml) and gradients were stored in –80°C. Prior to use, gradients were thawed overnight at 4°C. A 6–7 mm space from the top of the tube to the sucrose layer was ensured and 750 μl of the resuspended solution was layered drop by drop onto the gradient. Gradients were centrifuged at 49 100g for 16 to 17 h at 4°C in an SW32Ti rotor (Beckman). Gradients were fractionated using a Brandel (UV) gradient fractionation system and 750 μl fractions were collected at a flow rate of 1.5 ml/min. The 40S fractions (∼4 ml total) and 60S fractions (∼7.5 ml total) were pelleted by centrifugation at 63 000g for 20 h at 4°C in a Type 80 Ti rotor (Beckman Coulter) as described above. The subunit pellets were resuspended in storage buffer (30 mM HEPES pH 7.3, 100 mM KCl, 2.5 mM Mg (OAc)_2_, 2 mM DTT and 6% w/v RNase-free sucrose). Subunits were diluted 1:500 or 1:1000 in nuclease-free ultrapure water and OD 260 nm was measured (Agilent Cary 60 UV/VIS spectrophotometer). Subunit concentrations were calculated as described previously such that 1 *A*_260_ unit corresponds to 25 pmol of 60S and 50 pmol of 40S respectively (54,55). Subunits were aliquoted on ice in RNase-free tubes and frozen in liquid N_2_ and stored at −80°C. The 60S and 40S subunits were assessed by Coomassie staining and by western blotting using anti-uL14 (RPL23) (Bethyl) and anti-RPS10 (Santa Cruz Biotechnology) antibodies diluted in 1× TBS-T buffer (1:1000, overnight) as described above.

### Secondary structure determination using circular dichroism

CD measurements were performed using a Chirascan V100 spectrometer (Applied Photophysics Inc.) A nitrogen fused set up with a cell path of 10 mm was used to perform the experiments at 20°C. All CD traces were obtained between 200–260 nm, and background corrected using filtered CD reaction buffer (100 mM NaF, 1mM TCEP–HCl, and 5 mM Tris pH 7.5). All eIF6 proteins were first diluted to 12.5 μM in eIF6 storage buffer (50 mM HEPES pH 7.5, 200 mM NaCl, 5 mM β-mercaptoethanol and 10% glycerol) and then diluted into 3 ml CD reaction buffer to a final concentration of 0.6 μM. 10 scans were collected and averaged per experiment using 1 nm step size and 1 nm bandwidth. Variable temperature CD was captured by monitoring the molar ellipticity at 223 nm from 20°C to 90°C in 2.5°C increments. Thermal melt data was fit to a two-state unfolding model to obtain melting temperature (*T*_m_) (56).

### Subunit joining assay and negative-stain electron microscopy (EM)

The reactions for subunit joining assay were set up as described previously (9) except reactions were analyzed by negative EM. For the subunit joining assay, all reaction mixtures (50 μl) contained equal molar amounts (12 nM) of both 60S and 40S subunits in binding buffer (20 mM HEPES pH 7.3, 100 mM KCl, 1.5–10 mM Mg(OAc)_2_, 1 mM DTT, 1.5% glycerol). 20× molar excess eIF6 (280 nM) was pre-incubated with the 60S subunits for 5 min at 30°C in low (1.5 mM) Mg^2+^ binding buffer. Then, 40S was added in high Mg^2+^ binding buffer (10 mM final concentration) and incubated for 5 min at 30°C to allow for 80S formation. Reaction mixtures were immediately stained for EM imaging. For negative-stain EM, 20 μl of the subunit joining reaction mixtures were applied to plasma-cleaned carbon-coated 200 mesh copper grids (30 s using a Gatan Solarus 950). After 1 minute incubation, the grids were washed 5× with double-stilled water and stained with 0.75% uranyl formate for 2 min. The grids were then blotted with filter paper to remove excess stain, air-dried, and imaged using a JEOL JEM-1400 120 kV transmission electron microscope (TEM) equipped with an AMT NanoSprint15 Mk-II 15-megapixel camera. EM was performed at the Washington University in St. Louis cellular imaging core.

### Cell culture

HCT116 cells (human colorectal carcinoma line) (ATCC) were cultured as described previously (37,57). HCT116 cells were maintained in McCoy's 5A medium supplemented with 10% fetal bovine serum (FBS), and 100 units/ml penicillin and 100 μg/ml streptomycin (Gibco). Cells are routinely ensured to be negative for mycoplasma contamination using the mycoplasma detection kit (ATCC). For western blotting, the cells were washed twice in PBS and lysed in mammalian cell lysis buffer (MCLB) (50 mM Tris–Cl pH 8.0, 5 mM EDTA, 0.5% Igepal, 150 mM NaCl) that was supplemented with the following inhibitors just before lysis: 1 mM phenylmethylsufonyl fluoride (PMSF), 1 mM sodium fluoride, 10 mM β-glycerophosphate, 1 mM sodium vanadate, 2 mM DTT, 1× protease inhibitor cocktail (Sigma–Aldrich), 1× phosphatase inhibitor cocktail (Santa Cruz Biotechnology). Lysates were rocked for 15 min at 4°C followed by centrifugation at 14 000 rpm for 10 min at 4°C. Blots were probed with anti-eIF6 antibody (Santa Cruz Biotechnology, sc-390441) (1:1000, overnight) or anti-βTubulin antibody (Cell Signaling, 2128) (1:1000, overnight) diluted in TBS-T buffer.

### Cloning and transfections

To clone uL14 (RPL23), human uL14 transcript was PCR amplified using cDNA template and cloned into pCMV-Myc plasmid (Clonetech). The 8 or 20 residue C-terminal deletion mutations in pCMV-Myc-uL14 were generated by using Q5 site-directed mutagenesis kit (New England Biolabs) using forward and reverse primers (Supplementary Table S2). Gene sequences used in this study were confirmed by Sanger sequencing (Azenta-Genewiz). For transfections, 1.8 × 10^6^ HCT116 were plated per 60-mm dish and grown up to 70% confluency. Cells were transfected with 4 μg of plasmid DNA and 20 μl of Lipofectamine 2000 (Invitrogen) per 60-mm dish. Twenty six hours later, cells were washed and lysed in MCLB buffer supplemented with inhibitors as indicated above.

### Immunoprecipitation

For immunoprecipitation (IP) of endogenous eIF6, cells were lysed in MCLB buffer supplemented with inhibitors as indicated above. 700 μg of total protein in 500 μl of MCLB buffer supplemented with inhibitors was used for IP. For IP of Myc-uL14, 850 μg of total protein was suspended in a final volume of 500 μl of MCLB buffer supplemented with inhibitors. For IP, protein A/G plus agarose beads (Santa Cruz Biotechnology) were washed three times with cold MCLB buffer. Lysates were precleared with 20 μl of beads and 1 μl of normal mouse IgG (SCBT, 025) for 30 min at 4°C on a rotator. Precleared lysates were collected by centrifugation at 3500 rpm for 3 min at 4°C and incubated with 20 μl beads and 2.2 μg of anti-Myc-tag antibody (Santa Cruz Biotechnology, sc-40), anti-eIF6 antibody (Santa Cruz Biotechnology, sc-390441) or normal mouse IgG (sc-2025) and rotated overnight at 4°C. Unbound lysate was removed by centrifugation at 3000 rpm for 5 min at 4°C and beads were washed three times with cold MCLB buffer. Beads were re-suspended in 30 μl of MCLB buffer and eluted by boiling in 1× Laemmli buffer. Western blotting was performed as described above. Blots were probed with anti-eIF6 (1:1000; Santa Cruz Biotechnology, sc-390441), anti-eIF6 (1:1000; Cell Signaling, 3833), anti-Myc (1:1000; Cell Signaling, 2278), anti-uL14 (RPL23) (1:1000; Bethyl, A305-008A), and anti-βTubulin antibodies (1:1000; Cell Signaling, 2128) diluted in TBS-T buffer. (Note: Sequestration of the N-terminal tag of eIF6 by binding to affinity beads or antibodies can interfere with protein-protein interactions depending on the experimental set-up.)

### Cell viability assays

5000 HCT116 cells expressing eIF6-WT or eIF6-Y151A mutant were cultured per well of a 96-well plate in McCoy's culture medium without phenol red supplemented with 10% FBS and 1× penicillin–streptomycin solution. For serum starvation, 7000 HCT116 cells were cultured per well of a 96-well plate in McCoy's culture medium supplemented with 10% FBS and after overnight incubation, cells were washed twice with 1× PBS, and cultured in McCoy's medium without phenol red supplemented with 0.1% FBS and 1× penicillin–streptomycin solution. 20 μl of MTS solution (CellTiter 96^®^ Aqueous One solution reagent, Promega) was added to each well, and the plates were incubated for 1 h. Absorbances were read at 490 nm (Synergy H1, BioTek). To obtain background-corrected absorbances, average absorbance values of the control wells containing media and MTS only were subtracted from all other absorbances. Trypan blue exclusion test was performed as described before (37). Apoptosis was measured using the Caspase-Glo® 3/7 assay (Promega). Cells were seeded at a density of 1 × 10^4^ in triplicates in 96-well black polystyrene microplates (Corning). After 24 h of incubation at 37°C,  100  μl of caspase-Glo reagent (including caspase-Glo substrate and caspase-Glo buffer) was added to each well and incubated for  90  min at RT in dark. Luminescence was measured using a multi-mode microplate reader (Synergy H1, BioTek). To obtain background-corrected absorbances, average absorbance values of media with reagent only were subtracted from all other absorbances.

### CRISPR/cas9-mediated gene editing

*eIF6^Y151A/Y151A^* and *eIF6^N106S/N106S^* homozygous mutants were generated at the Genome Engineering and IPSC Center (GEIC) at Washington University in St. Louis. The Y151A and N106S point mutations were introduced by nucleofecting HCT116 cells with a synthetic guide RNA (gRNA)/Cas9 ribonucleoprotein complex along with a single stranded deoxyoligonucleotides (ssODN). gRNAs were selected based on off-target analysis performed by GEIC-specific algorithms. To generate the Y151A mutation, the gRNA recognition site is 5′-AGTAGCTTCCTACTAGCACC*NGG*, with the PAM site italicized, and the ssODN has the following sequence with two phosphorothioate bonds at each end: 5′- gacagaagaaattctggcagatgtgctcaaggtggaagtcttcagacagacagtggccgaTcaggtActagtGggaagcGCTtgtgtcttcagcaatcagggagggctggtgcatcccaagacttcaattgaagaccaggatg. To generate the N106S mutation, the gRNA recognition site is 5′-ggccacgtagtcattgcaggNGG and ssODNs have the following sequences: 5′acagcctcccagacacagtgcagattaggcgggtggaggagcggctctcagccttgggcaGtgtcacTacTtgcaatgactacgtggccttggtccacccagacttggacagggtgaggcagcccaacttg, 5′acagcctcccagacacagtgcagattaggcgggtggaggagcggctctcagccttgggcaGtgtcacAacAtgcaatgactacgtggccttggtccacccagacttggacagggtgaggcagcccaacttg. Synthetic gRNAs and ssODNs in ultramer format were purchased from IDT. The transfected pools of HCT116 cells were analyzed by using next generation sequencing for knock-in rate, and single-cell clones were obtained by sorting on a Sony sorter and screened by using next generation sequencing. Positive clones were expanded, and genotype confirmed prior to cryopreservation. All clones were negative for mycoplasma contamination and authenticated as HCT116 cells by STR profiling.

### 2D-gel electrophoresis

HCT116 cells were grown to 70–80% confluence and washed twice in PBS and collected in mammalian cell lysis buffer as described above. 2-D DIGE and Protein ID was performed by Applied Biomics, Inc (Hayward, CA). The protein extract was solubilized in 2D lysis buffer (30 mM Tris–HCl pH 8.8, containing 7 M urea, 2 M thiourea and 4% CHAPS. Protein concentration was measured using Bio-Rad protein assay method. For Cy-Dye labeling, 30 μg of protein was mixed with 1.0 μl of diluted Cy2, and kept in dark on ice for 30 min. The labeling reaction was stopped by adding 1.0 μl of 10 mM Lysine to each sample, and incubating in dark on ice for additional 15 min. The labeled samples were then mixed 150 μg unlabeled protein. The 2× 2D sample buffer (8 M urea, 4% CHAPS, 20 mg/ml DTT, 2% pharmalytes and trace amount of bromophenol blue), 100 μl destreak solution and Rehydration buffer (7 M urea, 2 M thiourea, 4% CHAPS, 20 mg/ml DTT, 1% pharmalytes and trace amount of bromophenol blue) were added to the labeling mix to make the total volume of 250 μl. The labeled samples are mixed then loaded into strip holder.

*IEF and SDS-PAGE:* After loading the labeled samples, IEF (pH3-10 Linear) was run following the protocol provided by GE Healthcare. Upon finishing the IEF, the IPG strips were incubated in the freshly made equilibration buffer-1 (50 mM Tris–HCl pH 8.8, containing 6 M urea, 30% glycerol, 2% SDS, trace amount of bromophenol blue and 10 mg/ml DTT) for 15 min with gentle shaking. The strips were subsequently rinsed in freshly made equilibration buffer-2 (50 mM Tris–HCl pH 8.8, containing 6 M urea, 30% glycerol, 2% SDS, trace amount of bromophenol blue and 45 mg/ml iodoacetamide) for 10 min with gentle shaking. Next, the IPG strips were rinsed in the SDS-gel running buffer before transferring onto 12% SDS-gels. The SDS-PAGE were run at 15°C until the dye front bled out of the gels. Gel image was scanned immediately following the SDS-PAGE using Typhoon TRIO (GE Healthcare). The scanned images were then analyzed by Image Quant software (version 6.0, GE Healthcare). After scanning, the proteins on the gel were transferred to Immobilon PVDF membranes (EMD Millipore, Billerica, MA) at 400 mA for 2.5 h. Upon completion of the transfer, the membrane images were immediately scanned using Typhoon TRIO. For the western blot, membranes were blocked in 5% BSA for 4 hrs with shaking. The membranes were then incubated overnight with shaking in primary antibody (anti-eIF6 antibody, Santa Cruz Biotechnology) in TBS-T buffer. The membranes were washed 4 times, 10 min each, with shaking in TBS-T buffer. The membranes were then incubated using Cy3 and Cy5-conjugated secondary antibody at a dilution of 1:2000 in TBS-T buffer with shaking for 2 hrs. The membranes were then washed 6 times with shaking, 10 min each, in TBS-T buffer. The membranes were scanned by Typhoon TRIO. The scanned images were then analyzed by Image Quant software (version 6.0, GE Healthcare).

## RESULTS

### Purification of recombinant human eIF6

Milligram quantities of eIF6 are required to perform detailed biophysical and structural characterization. Therefore, we first optimized the purification methodology to enhance the yield of recombinant full-length human eIF6. Previous studies have indicated that the overproduction of recombinant human eIF6 in *Escherichia coli* is limited due to poor solubility (2–5%) and results in low μg/ml yield of human eIF6 (36,58). Similarly, purification and yield of full-length *Saccharomyces cerevisiae* Tif6 from *E. coli* is hindered by the disordered nature of last 20 amino acid residues in the C-terminus (34). Removal of the last 20 residues enhanced the stability and yield of Tif6 (34). Therefore, we tested if deletion of the last 20 amino acid residues in the C-terminus of human eIF6 had a similar effect on protein yield. Deletion of the C-tail did not enhance the stability or solubility of eIF6 (Figure 1B). This behavior is distinct from yeast Tif6 and suggests that structural contributions of the C-terminal 20 residues in eIF6 may diverge between lower and higher eukaryotes.

Co-expression of a cocktail of five (5-CH: DnaK-DnaJ-GrpE, GroES, GroEL) bacterial chaperones improved the solubility of eIF6 (Supplementary Figure S1A). However, the gains from this strategy to improve yields of human eIF6 were nullified by the additional purification steps that were needed to eliminate the five chaperones (Supplementary Figure S1A). Furthermore, co-expression of the C-terminal deletion mutant of human eIF6 along with the five chaperones did not further improve the solubility of eIF6 (Supplementary Figure S1B). Thus, to limit the number of chaperones used, we systematically tested the chaperone combinations that were commercially available. We found that expression of the three chaperones (3-CH: GroES, GroEL, TF) that included the TF chaperone also enhanced solubility of eIF6. However, we still obtained low yields of eIF6 due to the additional purification steps that were needed to remove all three chaperones. However, co-expression of just the TF chaperone was sufficient to enhance solubility of human eIF6 and it simplified the purification steps needed to eliminate just one chaperone and resulted in better protein yields (Supplementary Figure S1A and C).

We next optimized the purification method for human eIF6 using affinity chromatography (Figure 1C). Interestingly, an array of proteins coelute with human eIF6 that could potentially be ribosomal factors. Subsequent fractionation of this complex using a Heparin column results in separation of human eIF6 from all other impurities and yields ≥98% pure full-length human eIF6 with high concentration of up to 5 mg/ml (Figure 1C). Using this methodology, we also obtained high yield of yeast Tif6 without the need to delete the terminal 20 residues (Supplementary Figure S1D). The identity of full-length eIF6 was further confirmed with an anti-His-antibody that recognizes the N-terminus of eIF6 and an anti-eIF6 antibody that recognizes the C-tail of eIF6 (Figure 1D). These results were further confirmed by mass spectrometry (data not shown). The recombinantly purified human eIF6 is active as it interacts with 60S in sucrose density gradient fractionation analysis (Figure 1E). We further confirmed the anti-association activity of eIF6 using the subunit joining assay. We verified the purity of the isolated human 60S and 40S subunits by probing for ribosomal factors that were specific to each subunit (Supplementary Figure S1E). Negative EM images showed that 60S and 40S subunits predominantly remain dissociated at lower Mg^2+^ concentrations (Figure 1F) and associate to form 80S only in the presence of higher Mg^2+^ concentrations (Figure 1G). However, the association of subunits is inhibited by pre-incubation of 60S with eIF6 (Figure 1H), which further indicates that the purified eIF6 is active. Successful overproduction and purification of milligram quantities of active human eIF6 enabled us to investigate the binding and conformational dynamics in the absence or presence of uL14.

### Residues in interface 1 contribute differentially to uL14–eIF6 interaction

**Figure 2.**
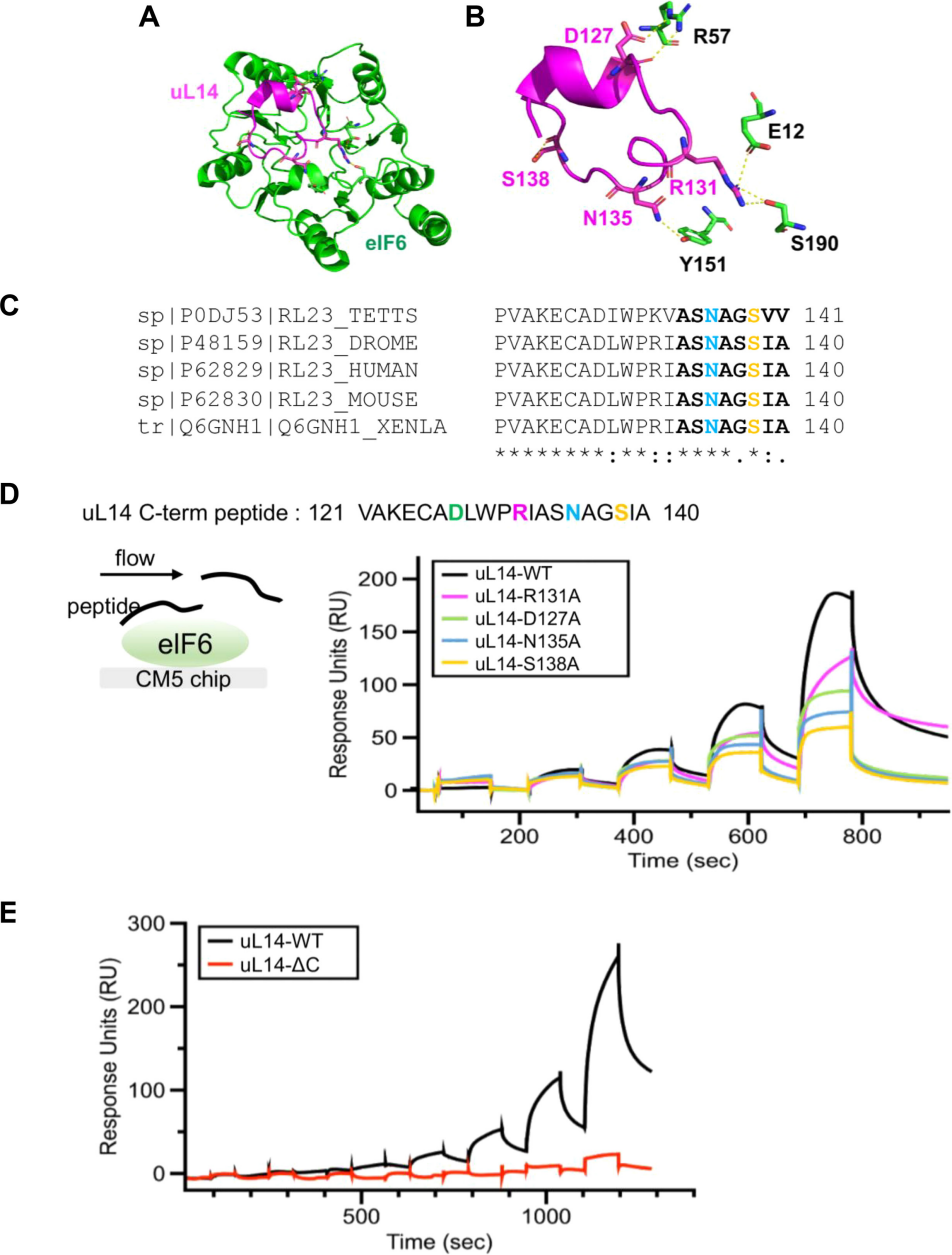
Residues in interface 1 contribute differentially to interactions between eIF6 and uL14. (**A** and **B**) Structures highlight the location of interface 1 and the contacts between eIF6 and uL14 (RPL23) (PDB code: 6LU8). (**C**) Analysis of the uL14 sequences (last 21 residues) from various eukarya show a high degree of conservation in the C-terminus of uL14. The terminal 8 residues in uL14 (bold) and the N135 (blue) and S138 (orange) residues are highlighted in the sequence. Sequences were aligned using Clustal Omega. Asterisk (*), colon (:) and dot (.) indicate identical residues, conserved and semi-conserved residues respectively. (**D**) Surface plasmon resonance experiments were performed by attaching eIF6 onto the CM5 chip and sequentially injecting increasing concentrations of uL14 peptide. Proportional binding and dissociation are observed as a function of peptide concentration. Experiments were performed with peptides carrying various mutations as denoted. The mutations result in loss of interaction of eIF6 to varying degrees, with the most severe loss of binding observed for the S138 to A substitution. (**E**) In SPR analysis, deletion of the last eight amino acids in the uL14 peptide results in complete loss of eIF6 binding.

Structural studies indicate that eIF6 interacts with 60S through direct contacts with uL14 (RPL23) and is positioned proximal to RPL24 and the sarcin-ricin loop (SRL) (11,35,59) (Figure 1A). The majority of these contacts are between eIF6 and a short helix at the C-terminus of uL14 (Figure 2A). These contacts are highly conserved and observed in 60S–eIF6 structures in other organisms. However, it has never been tested if the C-terminus of uL14 is sufficient for interaction with eIF6. Furthermore, the role of the individual contacts in promoting 60S binding to eIF6 are also unknown. This knowledge is required to probe this binding interface as a potential target for developing small molecule inhibitors against eIF6. In the human eIF6–60S structure, four residues in uL14 (D127, R131, N135 and S138) make key contacts with eIF6 (Figure 2B). These four residues and especially the terminal 20 residues in uL14 are highly conserved across species (Figure 2C). R131 forms a network of side chain interactions with E12 and S190. S138 makes backbone interactions within uL14 (Figure 2B). D127 and N135 interact with R57 and Y151 of eIF6, respectively (Figure 2B). However, in the cryo-EM structures that depict the nucleoplasmic-state A and cytoplasmic-state B of pre-60S subunits, the contacts between Y151 and uL14 are varied, which indicates the dynamic nature of these interactions (Supplementary Figure S2A–C). Therefore, to delineate the contributions of specific residues in the C-terminus of uL14 towards complex formation between uL14 and eIF6, we performed binding experiments using SPR.

For SPR studies, full-length elF6 was immobilized to the surface, and the C-terminal peptide of uL14 (aa 121–140) was used in the fluid phase (Figure 2D). We found that the uL14 peptide bound to immobilized eIF6 in a dose-dependent manner (Figure 2D). Association and dissociation profiles did not obey a 1:1 binding model suggesting a complex mechanism of interaction rather than non-specific binding of the peptide to the surface. The contribution of non-specific binding of the peptide to the surface was subtracted for each sensorgram. Importantly, alanine substitutions of key residues in the uL14 peptide R131, D127 and especially, N135 and S138 showed a significant reduction (∼50% reduction at 0.5 mM) in eIF6 binding (Figure 2D). It further highlighted the significance of the N135 and S138 for interaction with eIF6. Interestingly, N135 of uL14 interacts with Y151 in eIF6. Suppressor mutations in Y151 were previously identified to rescue the growth defect of *sdo1Δ* (SBDS homolog) and *efl1Δ* (EFL1 homolog) yeast strains that mimic the slow growth phenotype of SDS (60,61) (Supplementary Table S1). Thus, our SPR analyses indicate that Y151 is a key residue that is important for human eIF6 interaction with uL14 and explain the effect of Y151 mutation to rescue the eIF6-release defect seen in SBDS deficient yeast strains.

Somatic variants of eIF6 were recently identified in the hematopoietic cells of SDS patients and these variants were categorized as beneficial mutations that rescue the translational defect of SDS cells either by decreasing eIF6 levels or by disrupting the interactions between eIF6 and 60S (32,33). While the majority of the somatic mutations identified in SDS led to a marked loss of eIF6 expression, the predominant eIF6^N106S^ variant and *de novo* eIF6^R61L^ variant were expressed at levels similar to wild type eIF6 (32,62). However, the N106S and R61L mutations have been predicted by MD simulations to disrupt interactions with uL14 (32). In the cryo-EM structures of human eIF6 bound to pre-60S subunits, N106 of eIF6 also interacts with the backbone at A136 in the last 8 amino acids of the uL14 C-terminus (Supplementary Figure S2A). R61 on the other hand, does not make direct contacts with uL14, rather it stabilizes intra-eIF6 conformations through backbone interactions with several residues that in turn coordinate interactions of N106 and Y151 (Supplementary Figure S2A and B). All three residues- R61, N106 and Y151 are highly conserved in eukaryotes (Supplementary Figure S2D). Since N106 and Y151 makes multiple contacts with the terminal 8 residues in uL14, we aimed to determine if deletion of the terminal 8 amino acids in uL14 (uL14-ΔC) was sufficient for disrupting interactions with eIF6. The terminal 8 residues in the uL14 peptide also harbors the key S138 and N135 sites that were identified to be critical in Figure 2D. Deletion of the last 8 amino acids in uL14 leads to a complete loss in binding to eIF6 (Figure 2E). These experiments have uncovered that the terminal 8 residues in uL14 are critical for interaction with eIF6 *in vitro*.

**Figure 3.**
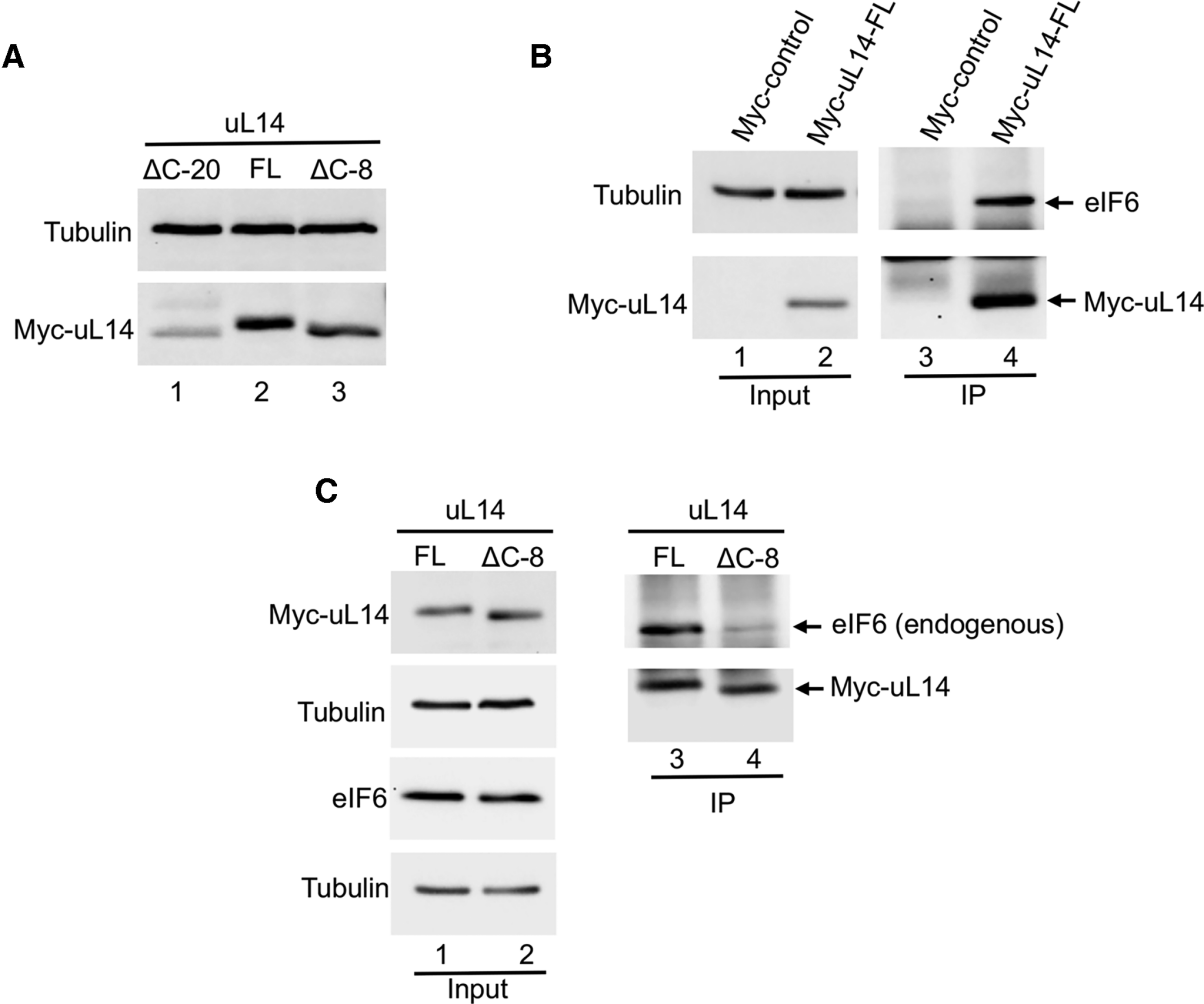
Terminal 8 residues in the C-tail of uL14 are critical for cellular interactions with eIF6. (**A**) Western blot shows expression of Myc-tagged uL14 lacking terminal 8 (ΔC-8) residues or 20 (ΔC-20) residues relative to the full-length (FL) uL14 in HCT116 cells. Blots were probed with anti-Myc antibody and Tubulin was used as loading control. Data is representative of three independent experiments. (**B**) Myc-tagged uL14 or Myc-empty vector control was immunoprecipitated from HCT116 cells. Western blot on the right (lanes 3 and 4) shows that immunoprecipitation with Myc-uL14 captures interactions with endogenous eIF6. Blots were probed with anti-Myc or anti-eIF6 antibodies. Blots on the left (lanes 1 and 2) represent the corresponding input. Tubulin was used as loading control. Data is representative of three independent experiments. (**C**) Myc-tagged uL14-FL or uL14-ΔC-8 was immunoprecipitated from HCT116 cells. Western blot (lanes 3 and 4) shows that only uL14-FL interacts with endogenous eIF6. Blots on the left (lanes 1 and 2) represent the corresponding input. Blots were probed with anti-Myc or anti-eIF6 antibodies. Tubulin was used as loading control. Data is representative of three independent experiments.

To determine if these terminal eight residues in uL14 are also critical for interactions with eIF6 in a physiological context, we expressed either Myc-tagged full-length uL14 (uL14-FL) or C-terminal deletion mutants lacking 8 residues (uL14-ΔC-8) or 20 residues (uL14-ΔC-20) in HCT116 cells. Interestingly, deletion of the 20 residues led to enhanced degradation of uL14 (Figure 3A). This suggests that the 20 residues in the C-tail of uL14 are critical for maintaining the stability and conformation of uL14 *in vivo*. Immunoprecipitation of Myc-uL14-FL showed that uL14 strongly interacts with endogenous eIF6 (Figure 3B and C). The specificity of immunoprecipitation was verified by using the Myc-empty vector control (Figure 3B). Immunoprecipitation of the uL14-ΔC-8 mutant showed that the mutation markedly disrupts interactions with eIF6 (Figure 3C). Both the uL14-ΔC-8 mutant and uL14-FL are expressed at similar levels indicating that the differences in immunoprecipitation are not due to variable expression (Figure 3C). Thus, these results showed that remarkably, even in a cellular environment, the terminal 8 residues in uL14 are critical for interactions with eIF6. These studies also provide a minimal binding region that can be targeted for the development of specific inhibitors.

### HDX-MS reveals global changes in eIF6 induced by binding to uL14

Structural information about 60S–eIF6 interactions is predominantly from cryo-EM studies that captured static snapshots of preformed complexes. However, real-time analysis of the changes in eIF6 structure upon binding to uL14 are lacking. We therefore initiated chemical cross-linking (XL-MS) and HDX-MS experiments to investigate conformational changes in eIF6 induced upon binding to uL14. The first step was to confirm the location of uL14 binding on eIF6. In cross-linking experiments, specific crosslinks are observed between the uL14 C-terminal peptide and residues in direct interaction interface 1 of eIF6 (Supplementary Figure S3A). Thus, our in-solution experiments show the uL14 peptide is binding at the expected interface on eIF6 as observed in structural studies. HDX-MS experiments were performed with human eIF6 in the absence and presence of the uL14 peptide. We have good sequence coverage of eIF6 based on pepsin peptides and can track changes in HDX for 180 of the 265 amino acids (∼70%) (Supplementary Figure S3B). For many of the regions, multiple overlapping peptides were identified (Supplementary Figure S3B). Data was collected as a function of time (30 s, 3 min, 30 min, 3 h and 24 h) and is described in terms of the kinetics and patterns of ΔHDX (net difference in deuterium uptake in the absence or presence of uL14 peptide).

**Figure 4.**
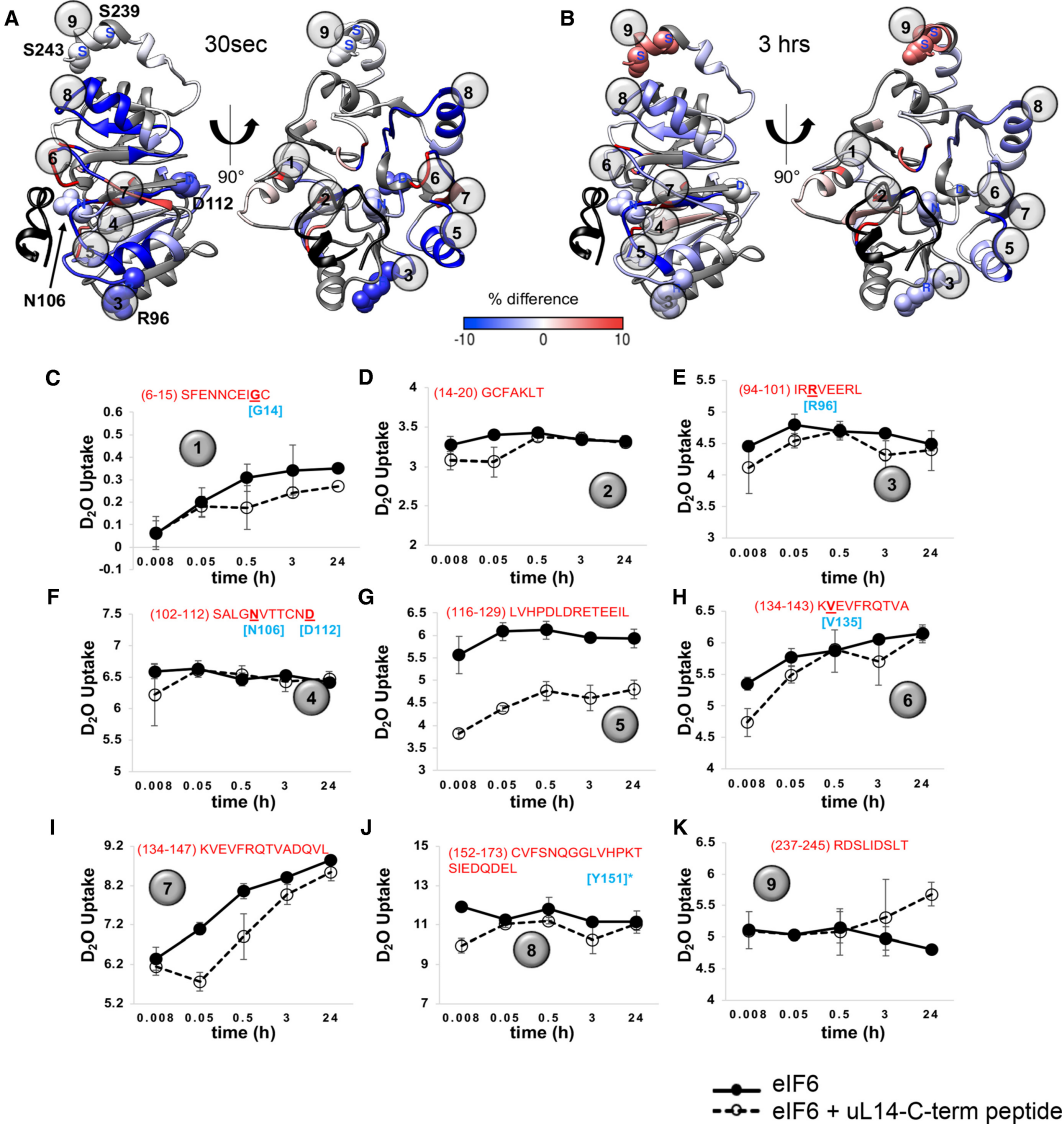
HDX-MS reveals dynamic changes in eIF6 upon uL14 binding. (**A** and **B**) are ΔHDX data mapped onto the structure of eIF6 from PDB ID 6LU8. ΔHDX denotes the scale of deuterium uptake or loss measured in the absence or presence of the uL14 peptide. The uL14 peptide bound in the structure is shown for reference (black). Numbers 1 to 9 denote the positions of the respective peptides shown in C to K. ΔHDX changes are seen in multiple regions in eIF6 including the C-terminal helix. (**C–K**) eIF6 peptides identified in HDX-MS analysis are shown. Data were collected as a function of time and deuterium uptake was measured in the absence or presence of the uL14 peptide. Sequence of the individual peptides are noted in each panel. Residues noted in cyan are SDS-patient associated mutations except for residue marked with an asterisk.

To construct a global landscape of the deuterium exchange and associated stability and dynamics of the hydrogen bonding network of eIF6 upon binding to uL14, we calculated ΔHDX in the absence and presence of peptide. The change in uptake at two data points (30 s and 3 h) was mapped on the structural model (Figure 4A and B). Increased deuterium uptake (red) indicates regions that are less protected (more exposed) in the presence of uL14 peptide. Conversely, change in the downward direction (blue) denotes decreased uptake (protected) upon peptide binding. ΔHDX comparison shows robust deuterium exchange in most regions of eIF6. Peptide level deuterium uptake curves are shown in Figure 4 and Supplementary Figures S4–S6. Supplementary Figure S6F shows that eIF6 protein assessed after 24 hours incubation with and without the uL14 peptide was intact and did not show any degradation or aggregation. Most of the peptides that contain amino acids in interface 1 of eIF6 and associated with SDS-disease mutations including residues that render eIF6 unstable (G14, R96, D112, V135) as well as residues that disrupt 60S interactions (N106 and Y151) have ΔHDX changes at early time points (Figure 4C–F, H–J), which further highlights the critical residues that dictate uL14 interactions. In terms of the kinetics of ΔHDX, both fast and slow exchange patterns are observed. We classify fast exchange as deuteration that has occurred during the early time points in our experiments (30 s–30 min). Slow exchange is deuterium uptake that occurs over longer periods (>30 min to 24 h). Differences in fast exchange are observed for multiple eIF6 peptides (e.g. Figure 4C–K) indicating a change in protection and/or dynamics. Decreases in HDX that are maintained across time scales are consistent with protection and the adoption of a stable conformation upon binding of uL14 (e.g. Figure 4G and I).

Interestingly, HDX differences are also observed in the C-terminal tail of eIF6 (Figure 4K). Several global phosphoproteomic studies have detected multiple phosphosites in the C-tail of human eIF6 especially at the S235, S239 and S243 residues (Supplementary Figure S7A and B). Two phosphosites (S231 and S233) have also been detected in the C-tail of yeast Tif6, so far (Supplementary Figure S7A and B) (63–68). Phosphorylation of S235 in the C-terminus of eIF6 has been shown to release eIF6 from the 60S (9). However, the mechanism of eIF6 release by the C-tail that is not in direct contact with the uL14 interface remains elusive. The ΔHDX observed in the C-tail upon binding to uL14 provides direct evidence for mechanical coupling between interface 1 and phosphosites on eIF6. This suggests an allosteric connection between the C-tail and interface 1.

Results show that uL14 binding at interface 1 induces changes in stability and dynamics within eIF6 and can be better interpreted by assigning defined states. If we were to subjectively assign *state^u^* as the conformation of unbound eIF6 (in the absence of peptide) and *state^b^* as the uL14-peptide bound, then transitions between the two states can be observed. In some regions a rapid and stable transition to *state^b^* is observed, as displayed by the constant ΔHDX over time (Figure 4G and I). This exchange pattern is consistent with long-lasting H-bond networks and formation of stable local structures. In some cases, the transition to *state^b^* is slow (Figure 4K and Supplementary Figure S4L) and such slow exchanges are a result of dynamic H-bond networks leading to solvent exposure and could be due to destabilization of the local structure. This infers slower remodeling rates due to conformational changes and potential allosteric processes (discussed below). Examples of such slow exchanges are observed in the disordered C-terminal region of eIF6 (Figure 4K). In many of the regions where uL14 peptide binding-induced changes in deuterium uptake are observed, increased protection is present (i.e. reduced deuterium uptake in the slow exchange regime, e.g. Figure 4C, G and Supplementary Figure S4D). This can be interpreted as protein surfaces that become buried upon uL14 peptide binding and are stable over the course of time (Figure 4H, I). If exchange is observed on the minutes to hours’ time scale, this suggests those peptide regions are transiently exposed to solvent and are therefore ‘less’ protected, hence more exposed, or dynamic (Figure 4K).

### Interface 1 disease mutations in eIF6 alter the secondary structure

**Figure 5.**
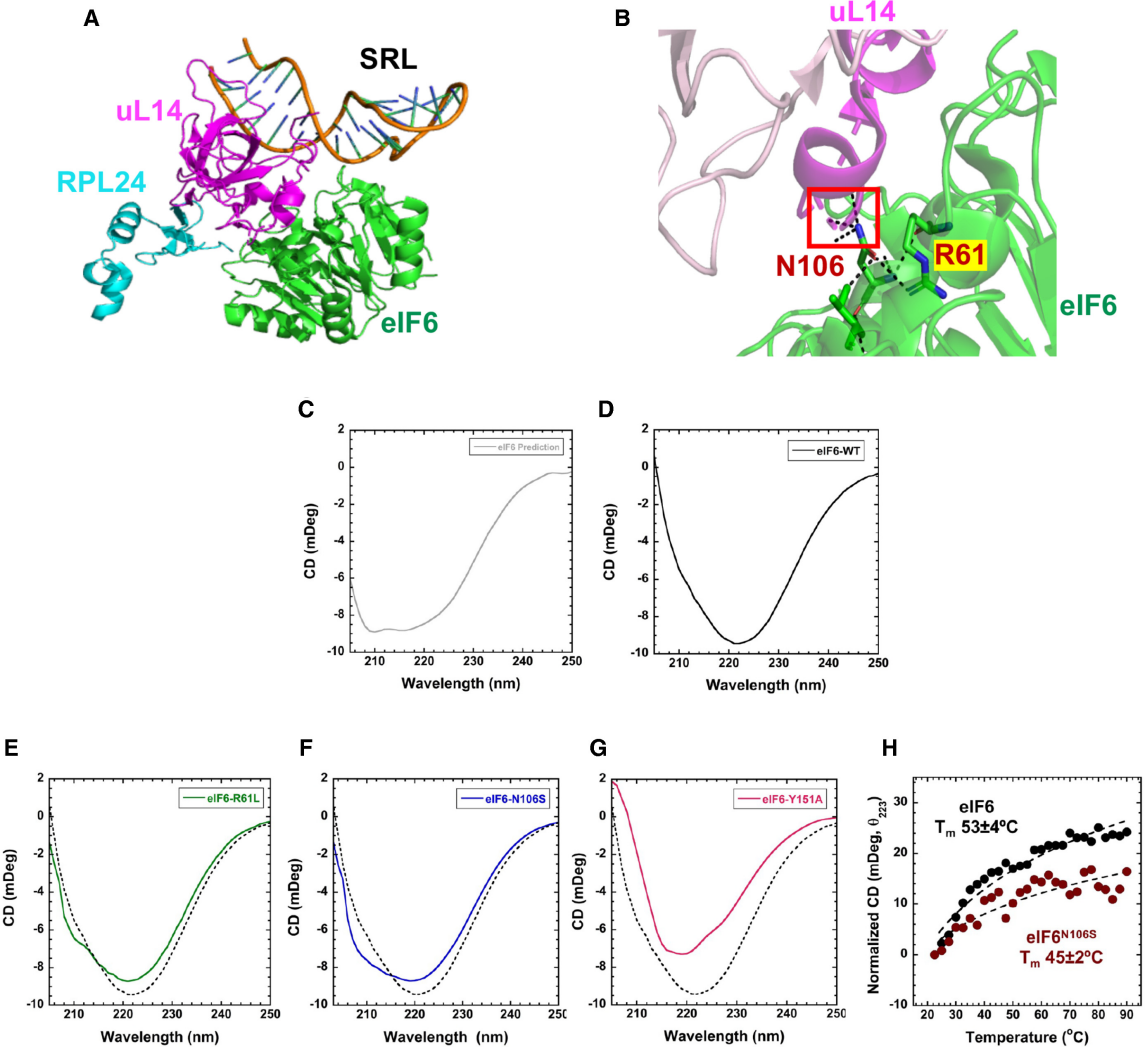
Secondary structure changes in SDS disease variants. (**A**) Positions of uL14 (magenta), RPL24 (blue), and the SRL in relation to eIF6. (**B**) Interactions mediated by N106 and R61 in eIF6 are depicted. (**C**) Predicted CD spectra from the structure of human eIF6 bound to 60S (PDB code 5AN9). In solution CD measurements of (**D**) eIF6-WT, (**E**) eIF6^R61L^, (**F**) eIF6^N106S^ and (**G**) eIF6^Y151A^ compared to eIF6-WT (dotted lines). Data represents three independent replicates. (**H**) Changes in CD signal at 223 nm were recorded as a function of temperature and yield *T*_m_ = 53 ± 4 and 45 ± 2 for eIF6 and eIF6^N106S^, respectively. Data is representative of three independent replicates.

The SDS-disease mutants of eIF6 reside directly in interface 1 or close to this region (Figure 5A and B). MD simulations have predicted that surface residues in interface 1 do not alter the overall structure of eIF6 as there is no significant effect on protein stability (32). However, mutations in this region are expected to result in a loss of interaction with 60S. Our ability to biochemically investigate human eIF6 allow us to experimentally test whether mutations in interface 1 cause structural perturbations. The cryo-EM structures of the 60S–eIF6 complex and crystal structures of eIF6 homologs from yeast and *Tetrahymena* show eIF6 as an ordered protein. However, our HDX-MS data show extensive changes in stability and the dynamics of the hydrogen bonding network occurring within eIF6 upon uL14 binding. Thus, in solution, the conformations of eIF6 are likely more dynamic. To gain structural insight into the solution structure of human eIF6, we performed CD analysis of the secondary structure. Simulation of the predicted CD profile for human eIF6 bound to 60S using PDBMD2CD yields a classical α-helical profile with double minima at 210 and 220 nm (Figure 5C). In solution CD measurements of unbound eIF6 show a slightly altered profile (Figure 5D). A strong minima is observed at ∼222 nm and reflects the α-helical content of eIF6. However, the predicted minima at ∼210 nm is overshadowed by an increase in β-sheet (and other) structural content. Analysis of the secondary structure content using BESTSEL shows ∼27% α-helix and ∼40% β-sheet (Supplementary Figure S8A). The comparison of the predicted secondary structure content from the structure of human eIF6 bound to 60S and the in-solution measurements of human eIF6 show identical α-helical content (27% each), but a considerable difference in the β-sheet content (10% versus 40%, respectively). Thus, the unbound eIF6 in solution (*state^u^*) exists in a slightly different conformation compared to when 60S bound (*state^b^*).

Next, we measured the CD profiles for mutant eIF6 proteins that were purified similar to WT (Supplementary Figure S8B) to capture the mutation-induced changes in secondary structure. We tested the two disease-variants: eIF6^R61L^ and eIF6^N106S^. The two mutant proteins show differences in the secondary structure compared to eIF6-WT (Figure 5D–G). eIF6^R61L^ and eIF6^N106S^ share similar α-helical content to eIF6-WT but show small increases in their β-sheet content. eIF6^N106^ was predicted to interact with 60S based on its position in interface 1. But MD simulations suggested that such a substitution will not alter the secondary structure of eIF6. Since *state^u^* displays a different secondary structure composition and the CD profile shows changes for the predominant eIF6^N106S^ mutation compared to wild type, we next tested the differences in their thermal stability (Figure 5H). In contrast to computational predictions, our CD analysis show that eIF6^N106S^ is thermally less stable (*T*_m_ = 45°C) compared to eIF6-WT (*T*_m_ = 53°C). Thus, mutations in interface 1 also have an influence on the secondary structure of eIF6 in the unbound state.

Since Y151 is one of the key residues in eIF6 that interacts with N135 of uL14, we also determined the effect of substituting Y151 with Alanine. eIF6^Y151A^ mutant shows a marked difference in CD spectra with a significant reduction of α-helical signature at 222 nm (Figure 5G). Apart from the key interaction with N135 in uL14, Y151 also mediates interactions with other residues within eIF6 and coordinates an extensive network of hydrogen bonding interactions that are core to the structure. Thus, Y151 plays an important structural role in *state^u^* of eIF6 (Supplementary Figure S9).

### eIF6-Y151 and N106 mutations disrupt binding to uL14 and inhibit proliferation of cancer cells

**Figure 6.**
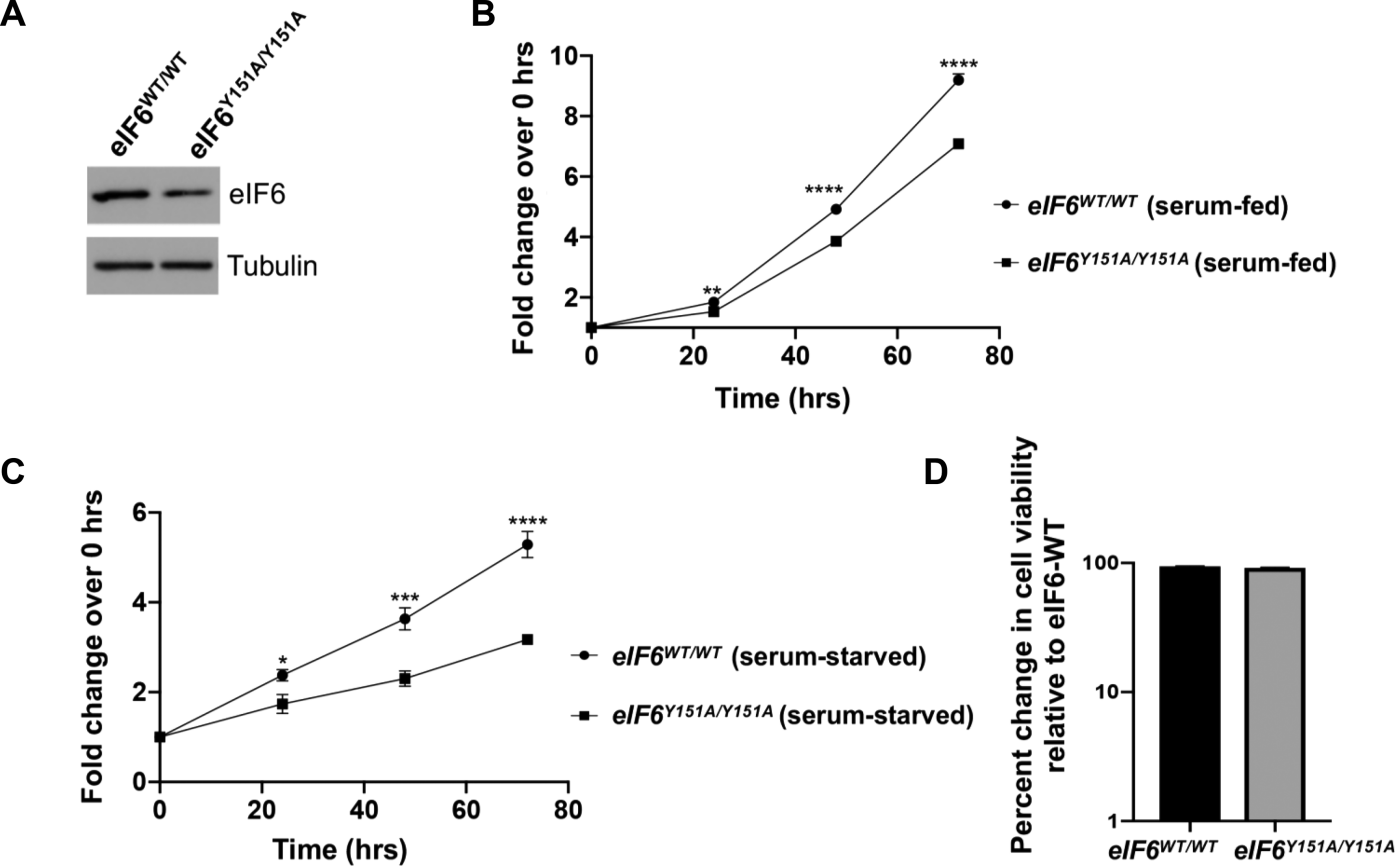
eIF6-Y151A mutation inhibits colonic cancer cell proliferation. (**A**) Western blot shows eIF6 expression in the isogenic *eIF6-WT* and *eIF6^Y151A/Y151A^*homozygous mutant HCT116 cells. Blots were probed with anti-eIF6 and anti-Tubulin (loading control) antibodies. Western blot is representative of three independent experiments. (**B**) Plot depicts the fold change in cell proliferation at 24, 48 and 72 h relative to 0 h as determined by MTS assay in serum-fed cells. Values represent standard error of the mean of three independent replicates. Asterisks indicate significant differences between eIF6-WT and eIF6-Y151A mutant at respective time points with *P* = 0.0019 at 24 h, *P*< 0.0001 at 48 and 72 h as determined by an unpaired two-tailed *t* test. (**C**) Plot depicts the fold change in cell proliferation at 24, 48 and 72 h relative to 0 hrs as determined by MTS assay in serum-starved cells. Values indicate standard error of the mean of at least three independent replicates. Asterisks indicate significant differences between eIF6-WT and eIF6-Y151A mutant at respective time points with *P* = 0.025 at 24 h, *P* = 0.0012 at 48 h and *P*< 0.0001 at 72 h as determined by an unpaired two-tailed *t* test. (**D**) Bar graph depicts percent change in cell viability of eIF6-Y151A mutant relative to eIF6-WT in serum-fed cells as determined by trypan blue exclusion test.

The significance of Y151 was further validated in cellular studies, where we assayed the effect of homozygous knock-in of Y151A mutant in HCT116 cells generated using CRISPR/Cas9-mediated genome editing. The Y151A mutant was expressed at slightly lower level than eIF6-WT (Figure 6A). Expression of the eIF6-Y151A mutant led to slower proliferation rates as shown by MTS assay (Figure 6B). eIF6-Y151A mutation also markedly sensitized cancer cells and inhibited cell proliferation in response to nutrient stress induced by serum starvation (Figure 6C). We did not observe any increase in cell death in mutant cells relative to wild type (Figure 6D). This marked effect of Y151A mutation in inhibiting growth of cancer cells provides the first genetic proof of concept that targeting the eIF6–60S interaction interface could be an effective strategy for cancer therapeutics.

**Figure 7.**
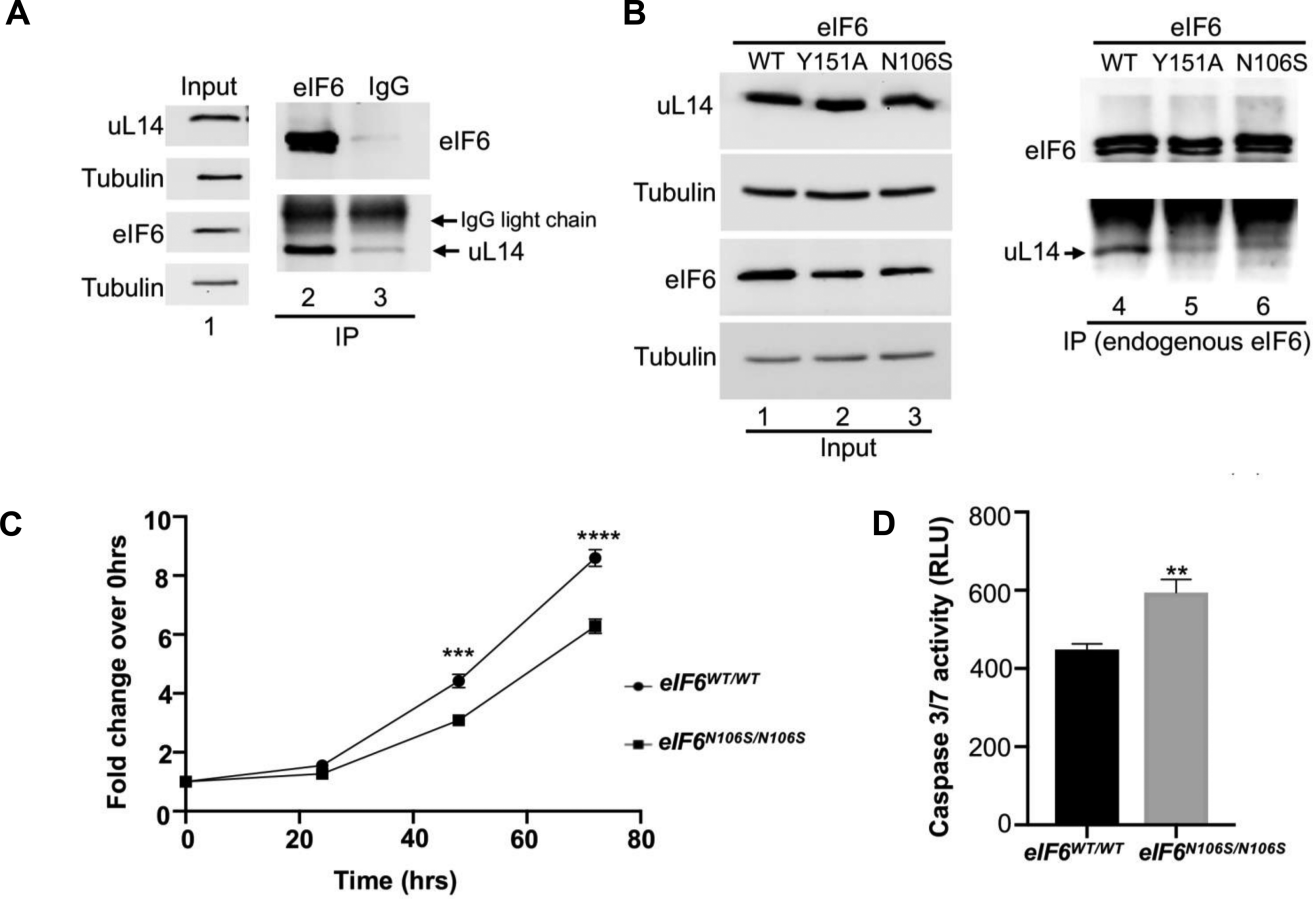
eIF6-N106S mutation disrupts interactions with uL14 and delays colonic cancer cell proliferation. (**A**) Immunoprecipitation of endogenous eIF6 using anti-eIF6 antibody or control mouse IgG from HCT116 cells. Lanes 2 and 3 in western blot show that immunoprecipitation of eIF6 captures interactions with endogenous uL14. (Note: uL14 and eIF6 migrate very close to the light chain IgG background seen for immunoprecipitation). Lane 1 represents the corresponding input assayed in lanes 2 and 3. Blots were probed with anti-uL14 and anti-eIF6 antibodies. Tubulin was used as loading control. Blots are representative of three independent experiments. (**B**) Homozygous knock-in of eIF6-N106S mutant was generated using CRISPR-genome editing of HCT116 cells. Western blot represents immunoprecipitation of endogenous eIF6 from eIF6-WT, eIF6-Y151A and eIF6-N106S expressing HCT116 cells. Lanes 4, 5 and 6 in western blot show that both eIF6-N106S and Y151A mutations disrupt binding to uL14. Lanes 1, 2 and 3 represent the input. Blots were probed with anti-uL14 and anti-eIF6 antibodies. Tubulin was used as loading control. Blots are representative of three independent experiments. (**C**) Plot depicts the fold change in cell proliferation at 24, 48 and 72 h relative to 0 hrs as determined by MTS assay in serum-fed cells. Values were corrected for background absorbance. Values represent standard error of the mean of three independent replicates and triplicate wells were assayed per experiment. Asterisks indicate significant differences between eIF6-WT and eIF6-N106S mutant at respective time points with *P* = 0.0001 at 48hrs and *P*< 0.0001 at 72 h as determined by an unpaired two-tailed *t* test. (**D**) Caspase3/7 activity was determined using Caspase3/7 glo assay. Bar graph displays data corrected for background absorbance. Error = SEM. Mean of three independent experiments with triplicate wells assayed per experiment were plotted. Asterisks indicate significant differences between eIF6-WT and eIF6-N106S with *P* = 0.0016 as determined by an unpaired two-tailed *t* test.

MD simulations and structural analyses have predicted that the predominant N106S disease mutation and Y151 mutation would disrupt interactions with uL14. However, this model was not experimentally probed. To address this, we immunoprecipitated endogenous eIF6 from HCT116 cells and captured the interactions between eIF6 and endogenous uL14 (Figure 7A). We observed that the immunoprecipitated eIF6 runs as a doublet. This doublet has been observed with very high levels of eIF6 associated with immunoprecipitation and is attributed to a gel effect due to the high cysteine content of eIF6 (9 cysteines for a 26 kDa protein) (37). Extensive characterization of the doublet and its discussion was included in our previous report (37). Specificity of immunoprecipitation was also verified using an IgG control (Figure 7A). To study the effect of N106S mutation on eIF6 interaction with uL14, we generated a homozygous knock-in of eIF6-N106S mutant (*eIF6^N106S/N106S^*) using CRISPR/Cas9-mediated genome editing in HCT116 cells. The eIF6-N106S mutant is expressed at a slightly lower level than eIF6-WT (Figure 7B). Remarkably, immunoprecipitation of the eIF6-N016S and eIF6-Y151A mutants showed that the mutants bind poorly to uL14 (Figure 7B). These results directly demonstrate that the predominant disease mutation of eIF6 (N106S) disrupts interactions with uL14 (Figure 7B). These results also substantiate the predisposition of SDS patients to harbor eIF6 mutations that disrupt interactions with uL14 to rescue the SDS phenotype.

Using the *eIF6^N106S/N106S^* line, we also determined the effect of mutation on the growth and viability of colonic cancer cells. Expression of the eIF6-N106S mutant markedly sensitized colonic cancer cells and inhibited cell proliferation similar to the eIF6-Y151A mutant (Figure 7C). A mild increase in apoptosis was also observed for the eIF6-N106S mutant (Figure 7D). This marked effect of both the N106S and Y151A mutations of eIF6 in inhibiting growth of cancer cells further highlights the significance of targeting the eIF6–60S interaction interface as an effective strategy for cancer therapeutics.

### The C-terminus of eIF6 offers a second regulatory interface 2 for uL14 interactions

**Figure 8.**
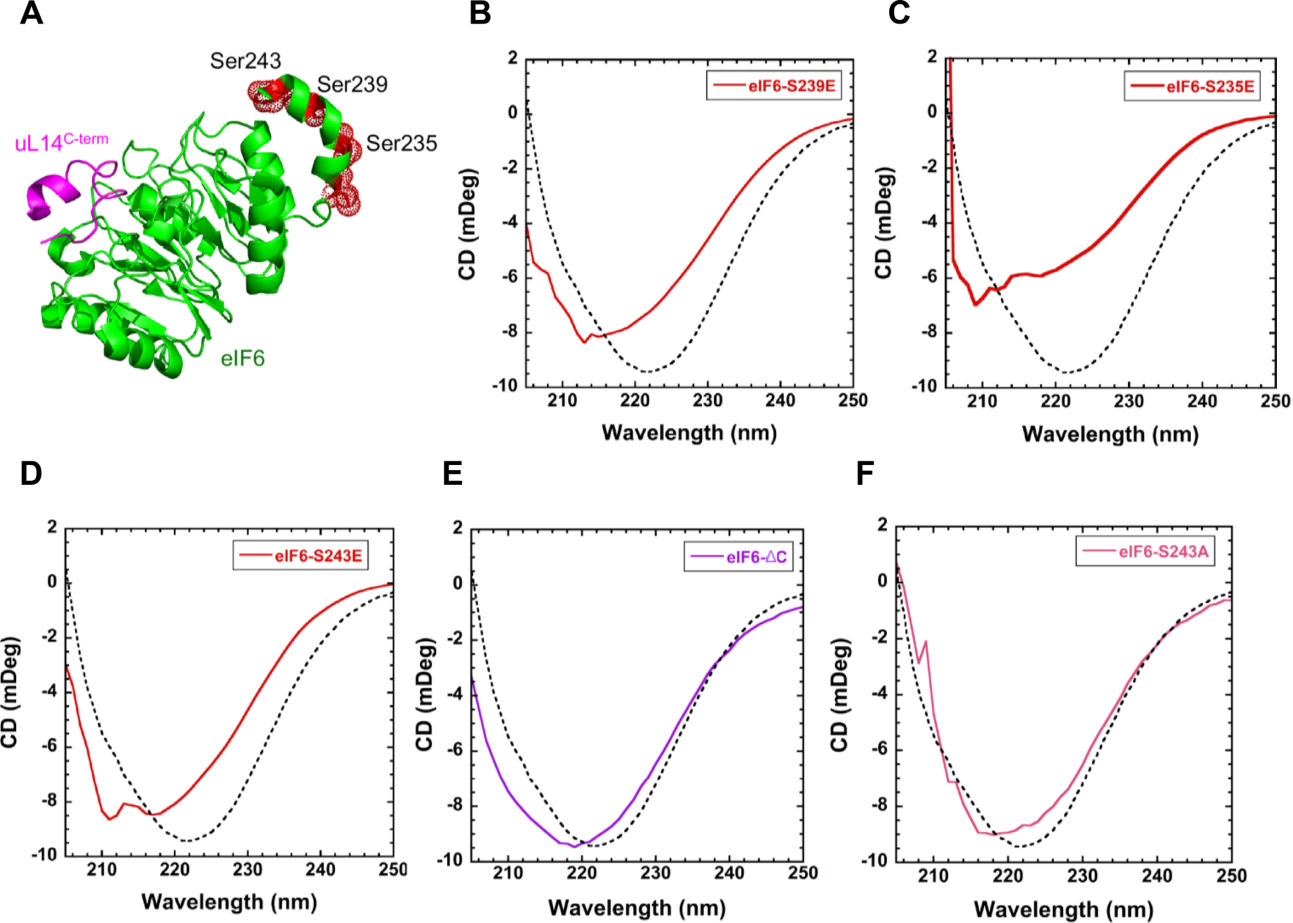
Phosphomimetic substitutions induce secondary structure changes in eIF6. (**A**) Positions of known sites of phosphorylation in eIF6 are shown in red (PDB code 5AN9). (**B**) In solution CD measurements of eIF6^S239E^, (**C**) eIF6^S235E^, (**D**) eIF6^S243E^, (**E**) eIF6^ΔC^ and (**F**) eIF6^S243A^ compared to eIF6-WT (dotted lines). Changes in secondary structure are observed for all the phosphomimetic substitutions. eIF6^ΔC^ shows a shift in the CD spectrum due to deletion but maintains the overall profile. eIF6^S243A^ shows minimal changes in secondary structure compared to wild type eIF6. Data represents three independent replicates.

The HDX-MS experiments uncovered allostery-driven structural changes in the C-tail of eIF6 upon binding to uL14 in interface 1 (Figure 4K). Several sites of phosphorylation have been identified in this region (Figure 8A), with S239 and S243 being the predominant sites detected in the phosphoproteomic studies as indicated in Supplementary Figure S7B. S/T239 and S/T243 sites are also highly conserved in both higher and lower eukaryotes (Supplementary Figure S7A). Our previous study also detected phosphorylation of the S239 and S243 sites in serum-starved cells and showed that phosphorylation can functionally regulate eIF6 (37). In addition, our 2D-gel analysis of endogenous eIF6 in colon carcinoma cells indicates extensive phospho-modification (Supplementary Figure S10 A and B). These results are consistent with previous 2D analysis of eIF6 in malignant pleural mesothelioma cells that showed extensive phosphorylation of C-tail of eIF6 that was lost upon phosphatase treatment (20). To better understand the effects of C-tail phosphorylation, we tested if substitution of phosphomimetic amino acids (S to E substitution) in the C-terminal region alter the secondary structure of eIF6. The phosphomimetic mutants were purified similar to WT (Supplementary Figure S10C). When negative charges are introduced in the C-terminus at the respective sites: eIF6^S239E^, eIF6^S235E^ and eIF6^S243E^ (Figure 8B, C and D), all phosphomimetic mutants show marked changes in the secondary structures compared to WT-eIF6. Deletion of the C-terminus (eIF6^ΔC^) does not change the profile of the CD spectrum but shifts the overall profile due to the loss of the residues (Figure 8E). As a control experiment, when S243 is substituted with an A, CD spectra are similar to WT-eIF6 (Figure 8F) indicating that the addition of charges rather than mutation of the residue contributes to the changes in CD spectra. These CD data indicate that phosphorylation at these residues can significantly influence the overall conformation of the non-60S bound state of eIF6 (*state*^u^). These data are in agreement with the ΔHDX changes observed in the C-terminus upon uL14 peptide binding. It is likely that when the specific sites are phosphorylated, the C-tail binds to other regions in eIF6 and influences the secondary structure. In support of this model, the eIF6 structure from *Chaetomium thermophilum* (Tif6) shows two sulfate ions bound to the flat surface (interface 2) away from the uL14 binding interface 1 (Supplementary Figure S11). Such intra-eIF6 interactions mediated by the negatively charged C-tail could influence the overall secondary structure and either modulate interactions with 60S or alter protein stability.

## DISCUSSION

**Figure 9.**
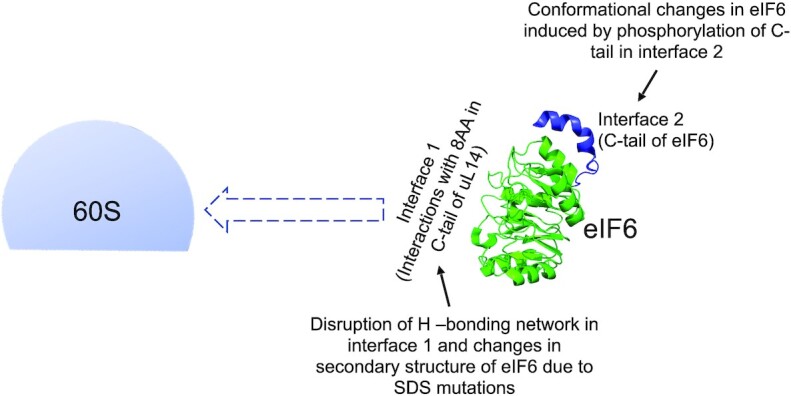
Model depicts the influence of interface 1 and 2 of eIF6 on 60S association. The key residues of interaction in interface 1 between eIF6 (PDB code 6LU8) and the terminal 8 amino acids in the C-tail of uL14 and conformational changes in disease variants influence the direct association of eIF6 with 60S and could influence the kinetics of eIF6 release from 60S. Phosphorylation of the C-tail of eIF6 in interface 2 and associated conformational changes of phosphomimetic mutants could also influence eIF6 interactions with 60S.

In this study, we show that eIF6 is conformationally dynamic and undergoes extensive conformational changes upon binding to the uL14 interface of 60S subunit. We also establish that the terminal 8 residues in the C-terminus of uL14 (RPL23) and especially N135 and S138 residues are critical for interaction with eIF6 both *in vitro* and *in vivo* (Figure 9). The positioning of these residues and their associated contacts are expected to be disrupted by the N106S mutation that is predominant in SDS patients. We now show that indeed the N106S mutation disrupts interactions with uL14 *in vivo*. This substantiates the poor binding of N106S mutant to the 60S (32,33). Mammalian eIF6 interacts with uL14 through pre-60S and 60S, but also as a free complex, which has been captured using co-precipitation studies similar to those shown in Figure 3C (69,70). More importantly, these uL14 interactions are essential for eIF6 binding to the pre-60S as depletion of uL14 markedly reduces eIF6 recruitment to the pre-60S (71). Thus, our data and previous structural studies indicate that the interactions between 60S and eIF6 occur primarily through uL14. There is an effort to therapeutically target eIF6 by screening for compounds that broadly target eIF6 and 60S interactions (72). In this study, by identifying the key residues of interaction, we now present a framework for a more selective target to screen for small molecule inhibitors.

In SDS hematopoietic cells, several truncating mutations of *EIF6* as well as a rare interstitial deletion in chromosome 20 that deletes *EIF6* were identified (32,33). In addition, several point mutants of *EIF6* were identified that were categorized as either *unstable mutants* that rendered eIF6 unstable and decreased eIF6 levels or *interaction-site mutants* that were predicted to disrupt interactions with 60S without decreasing eIF6 levels (32,33). Several common point mutations including the prominent R96W mutation render eIF6 unstable and these mutants are expressed poorly (32,33). However, the R61L mutation and the predominant N106S mutation are interaction-site mutants that are expressed at levels similar to WT but show reduced binding to the 60S subunit as shown by sucrose density gradient analysis (32). MD simulations and homology models have predicted the effects of mutations on the overall stability and conformation of eIF6 (32,33). For the N106S mutation, it was predicted that the sidechain of serine formed weaker H-bonds with the backbone of uL14 residues A133 and A136 or with R61 that weakens the H-bonding network and thereby disrupts the uL14-interaction interface (32). Since the simulations focused on uL14 interactions, we wanted to determine if these mutations also affected the overall conformation of eIF6. Here, through direct testing, our CD spectra show that the secondary structure of N106S mutant is significantly altered compared to WT. This indicates that besides the influence of the side chain of the substituted serine, the overall change in the N106S mutant conformation will further disrupt the H-bonding network in the interaction interface and weaken the binding of N106S to 60S.

Mutations of Y151 and N106 residues of eIF6 (Tif6) were identified among the gain of function alleles that suppressed the slow growth phenotype of *sdo1Δ* and *efl1Δ* yeast strains (60,61). Biallelic mutations in the *EFL1* GTPase are observed in SDS patients, although at a lesser frequency than *SBDS* mutations (17). Similar to SBDS mutations, the EFL1 mutations also impair the release of eIF6 from 60S subunits (17). The slow growth defect of the Efl1 deletion strain in yeast could be rescued by mutations in the Y151 and R61 residues in eIF6 (Supplementary Table S1). Mutations in both the Y151 and R61 residues were also observed in the yeast strains lacking *Sdo1* (Supplementary Table S1). Interestingly, as shown in Supplementary Table S1, several of the mutations that were found to rescue the slow growth phenotype in yeast *sdo1Δ* strain were also found in SDS patients. These studies highlight that the residues critical for eIF6 function are highly conserved across species. However, among all the mutations identified in yeast and patient studies, structural analyses indicate that mutation of these four key residues: N106, Y151, R57 and R61 are highly likely to disrupt direct interactions with uL14 (Supplementary Table S1). Through direct analysis of endogenous protein-protein interactions, in this study we now show that disrupting the N106 and Y151 residues markedly affect interactions with uL14.

Disruption of both the Y151 and N106 residues that are critical for binding to uL14, caused a marked inhibition of cancer cell proliferation under serum-fed state. These studies strongly suggest that targeting the uL14 interaction interface is an effective therapeutic strategy for cancers. Interestingly, the eIF6-Y151A also caused a profound inhibition of proliferation under conditions of nutrient deprivation. This is consistent with our previous study where we uncovered that eIF6 is important for cellular adaptation to nutrient stress and this role is conserved even in bacterial cells as shown by RsfS (RsfA) function that binds to the same uL14 (rplN) interface (37,43). These studies suggest that effect of eIF6 inhibitors could be enhanced by fasting. Future studies will assess the effect of targeting the eIF6–60S interaction interface on tumor progression *in vivo*.

Intriguingly, Y151 variants are yet to be identified in SDS patients, whereas N106S is a prominent mutation identified in SDS patients and yeast (Supplementary Table S1). Somatic mutations of eIF6 in SDS patients are heterozygous. This suggests that a partial loss of eIF6 function proportional to SBDS deficiency is sufficient to rescue cellular fitness and promote clonal evolution of eIF6 mutant cells. However, any further loss of eIF6 or further decrease in association of eIF6 with 60S is likely to switch the balance to translation inhibition and to an inhibition of ribosome biogenesis. A marked loss of eIF6 can lead to spurious 60S and 40S association and thereby hinder translation. It is thus possible that more severe mutations or homozygous mutations are not as favored for clonal selection in SDS patients as they could switch the balance from rescue of cellular fitness to inhibition of growth. This threshold effect could also explain the paradox of the strategy to target the eIF6–60S interaction interface in cancers to inhibit growth whereas targeting the same interface in SDS patients can be used to rescue the slow growth phenotype and cellular fitness. In addition, normal cells that exhibit haploinsufficiency of eIF6 (eIF6^+/−^ mice) do not display changes in basal translation rates but such partial loss of eIF6 is sufficient to inhibit tumorigenesis. Thus, a dosage threshold of inhibiting eIF6 should also be taken into consideration for therapeutic screening of compounds to target eIF6–60S interactions in SBDS mutant cells versus cancers and to minimize side effects. Future studies will probe the threshold-effect of targeting eIF6 based on the cellular context and diseased state.

Almost two decades ago, phosphorylation of S235 was shown to release eIF6 from 60S (9). However, the mechanism for how phosphorylation of the C-tail influences association with 60S without direct interactions with the uL14 interface has remained unknown. Here, we show for the first time the effect of the C-terminal residues on the overall conformation of eIF6 and the dynamic solvation of the C-tail upon binding to uL14 (Figure 9). This suggests that the overall changes in eIF6 conformation mediated by phosphorylation of the C-tail could alter the H-bonding network at the uL14 interface and potentially release eIF6 from 60S through allosteric regulation. However, it remains unclear as to how the C-tail contributes to the mechanism of release mediated by SBDS and EFL1 GTPase. It has to be noted that these analyses are based on *in vitro* systems and could change based on the physiological state of the pre-60S ribosomal particles *in vivo*.

Since phosphorylation of endogenous eIF6 at the S243, S239 and S235 sites has been captured by several phosphoproteomic studies, we attempted to synthesize phospho-specific antibodies targeted against the S235 and S239 sites. However, antibody generation has been quite challenging due to the high hydrophobicity of C-tail and has yielded non-specific antibodies with enhanced cross-reactivity with the non-phosphorylated form of eIF6. Future studies will optimize antibody production to better understand the contributions of the phosphorylated form. Given the marked effect of phosphorylation on eIF6 conformation, it is likely that phosphorylation permits release of eIF6 from nascent 60S or prevents re-binding of eIF6 to recycled 60S post-termination. Studies thus far indicate that the phosphorylated fraction as well as the specific sites of phosphorylation may vary based on the physiological context and future studies will aim to better understand the mechanism and context-specific regulation of phosphorylation.

## DATA AVAILABILITY

All data have been included in the manuscript.

## Supplementary Material

gkac1266_Supplemental_File
